# Hierarchical Self‐Assembly Molecular Building Blocks as Intelligent Nanoplatforms for Ovarian Cancer Theranostics

**DOI:** 10.1002/advs.202309547

**Published:** 2024-02-26

**Authors:** Shuo Li, Qingrong Chen, Qi Xu, Zhongyu Wei, Yongjin Shen, Hua Wang, Hongbing Cai, Meijia Gu, Yuxiu Xiao

**Affiliations:** ^1^ Department of Thyroid and Breast Surgery Zhongnan Hospital of Wuhan University Key Laboratory of Combinatorial Biosynthesis and Drug Discovery (Ministry of Education) School of Pharmaceutical Sciences Wuhan University Wuhan 430071 China; ^2^ Jiangsu Institute of Hematology National Clinical Research Center for Hematologic Diseases NHC Key Laboratory of Thrombosis and Hemostasis The First Affiliated Hospital and Collaborative Innovation Center of Hematology Soochow University Suzhou 215006 China; ^3^ Department of Gynecological Oncology Zhongnan Hospital of Wuhan University Hubei Key Laboratory of Tumor Biological Behaviors Hubei Cancer Clinical Study Center Wuhan 430071 China

**Keywords:** β‐galactosidase, hierarchical self‐assembly, ovarian cancer, photothermal therapy, polymers, theranostics

## Abstract

Hierarchical self‐assembly from simple building blocks to complex polymers is a feasible approach to constructing multi‐functional smart materials. However, the polymerization process of polymers often involves challenges such as the design of building blocks and the drive of external energy. Here, a hierarchical self‐assembly with self‐driven and energy conversion capabilities based on *p*‐aminophenol and diethylenetriamine building blocks is reported. Through β‐galactosidase (β‐Gal) specific activation to the self‐assembly, the intelligent assemblies (oligomer and superpolymer) with excellent photothermal and fluorescent properties are dynamically formed in situ, and thus the sensitive multi‐mode detection of β‐Gal activity is realized. Based on the overexpression of β‐Gal in ovarian cancer cells, the self‐assembly superpolymer is specifically generated in SKOV‐3 cells to achieve fluorescence imaging. The photothermal therapeutic ability of the self‐assembly oligomer (synthesized in vitro) is evaluated by a subcutaneous ovarian cancer model, showing satisfactory anti‐tumor effects. This work expands the construction of intelligent assemblies through the self‐driven cascade assembly of small molecules and provides new methods for the diagnosis and treatment of ovarian cancer.

## Graphical abstract

Inspired by biological self‐assembly in nature, a novel hierarchical self‐assembly polymer with fluorescence and photothermal properties is proposed. The self‐assembly process is activated under the specific action of enzymes, enabling dual‐mode liquid biopsy and in vitro selective imaging. The polymer achieves tumor ablation through photothermal treatment. The intelligent polymer self‐assembly platform enables the diagnosis and treatment of ovarian cancer.

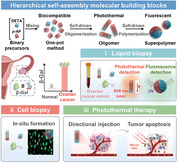

## 1 Introduction

**Figure 1:**
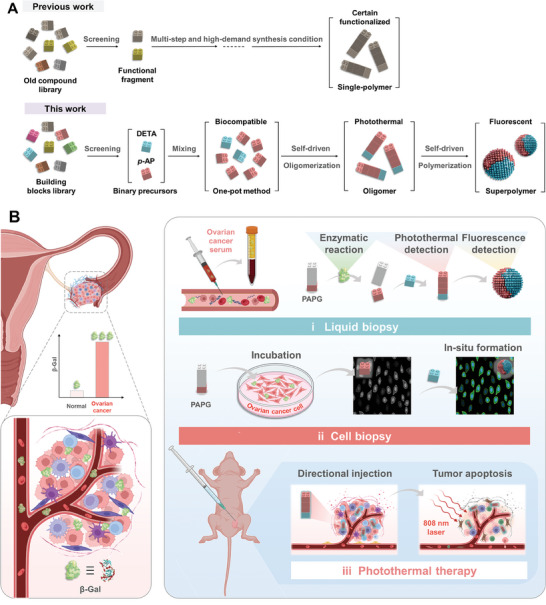
Overall concept of ovarian cancer theranostics based on self‐assembled polymers. A) Schematic illustration of the polymerization process of previous work and this work. B) Schematic illustration of self‐driven cascade polymers as versatile nanoplatforms for multi‐mode ovarian cancer precision theranostics.

Hierarchical self‐assembly from simple to complex is the most basic substance synthesis strategy in nature, which is of great significance for the in‐depth understanding of the assembly process from molecular, nano to macro scale.^[^ ^1^, ^2^, ^3^ ^]^ It also provides a feasible approach for the construction of advanced intelligent materials.^[^ ^4^ ^]^ Driven by various self‐assembly forces, multiple polymers with exquisite structures have emerged, which have great potential as functional biomaterials in disease therapy,^[^ ^5^ ^]^ drug delivery,^[^ ^6^ ^]^ chemical sensing,^[^ ^7^ ^]^ and catalysis.^[^ ^8^, ^9^ ^]^ However, previously reported polymers often require special molecular monomer designs (e.g., peptides,^[^ ^10^ ^]^ DNA sequences,^[^ ^11^ ^]^ and protein region^[^ ^12^ ^]^) or highly specific mechanisms to control intermolecular interactions (e.g., metal‐organic coordination,^[^ ^13^ ^]^ DNA hybridization,^[^ ^14^ ^]^ and antibody‐antigen specificity^[^ ^15^ ^]^) to trigger assembly.^[^ ^16^ ^]^ And these assembly processes are also subject to external physical factors, resulting in limited and high‐demand synthetic conditions (**Figure** **1**A).^[^ ^17^, ^18^ ^]^ We expect there to be a self‐assembly strategy, which regards molecule monomers as building blocks and uses minimal molecular units and fundamental forces as possible to overcome the limitations of the assembly process above and build advanced intelligent assembles.^[^ ^19^, ^20^ ^]^ Finding such molecular building blocks to expand and realize more complex applications of polymers is a key direction in the development of polymers.

Ovarian cancer is a perilous form of cancer that targets the ovaries and is widely known as the first malignant cancer in gynecology.^[^ ^21^, ^22^, ^23^ ^]^ In recent years, the number of ovarian cancer patients worldwide has increased year by year, and the majority of ovarian cancer patients are already at the advanced stage when first diagnosed, and the five‐year survival rate is less than 30%.^[^ ^24^ ^]^ Therefore, exploring new strategies to provide accurate diagnosis and effective treatment of ovarian cancer is critical. Theranostics for cancer is the integration of diagnosis and treatment and is considered the next generation of personalized medicine.^[^ ^25^ ^]^ Among various technologies, fluorescence imaging/sensing has excellent sensitivity and accuracy, and photothermal therapy has become the frontier of precision medicine against cancer due to its strong controllability and minimal invasive burden.^[^ ^26^, ^27^ ^]^ However, the majority of the nanomaterials only combine therapeutic agents with imaging probes, their diagnostic signals and therapeutic effects are always on.^[^ ^28^, ^29^, ^30^ ^]^ This inevitably leads to signal interference and unwanted side effects in normal tissues.^[^ ^31^ ^]^ Therefore, the ideal therapeutic agent should have the ability to activate signals only at the disease foci. The use of cancer‐specific overexpression enzymes to activate the opening of diagnosis and treatment not only provides valuable signal amplification, but also provides a feasible approach for precision diagnosis and treatment.^[^ ^32^, ^33^, ^34^, ^35^ ^]^

Our previous work focused on the assembly of cascade polymers mediated by the metal‐organic framework, which encourages us to simplify the hierarchical synthesis of polymers and expand them to ovarian cancer therapy.^[^ ^36^ ^]^ In this work, we build an unreported library of molecular building blocks and screen out a novel self‐driven cascade polymerization between *p*‐aminophenol (*p*‐AP) and diethylenetriamine (DETA). This polymerization relies solely on dynamic covalent bonds between these simple, low‐cost, and readily available molecular building blocks, and primary particle formation and functional sets can be integrated into a time‐gated hierarchical polymerization process. Further, we found that the formed oligomer (OM) has a robust photothermal effect, while later superpolymer (SP) with strong fluorescence emission is formed (Figure 1A). β‐Galactosidase (β‐Gal) is a hydrolase in the cellular lysosome that hydrolyzes lactose to galactose.^[^ ^37^ ^]^ Importantly, β‐Gal is highly expressed in ovarian cancer cells and therefore can be used as a specific biomarker for primary ovarian cancer, which inspires us to extend this time‐gated hierarchical polymerization process between *p*‐AP and DETA into the multi‐modal diagnosis and treatment of ovarian cancer.^[^ ^38^, ^39^ ^]^ Through the design of the enzyme‐substrate, these cascade polymers (OM and SP) can be formed in situ under the activation of β‐Gal in pathological environments, and their excellent fluorescence and photothermal properties enable molecular and cellular level diagnosis and further complete the photothermal ablation of solid tumors. Finally, the self‐driven cascade polymerization opens up a new path for the low‐cost in‐situ manufacturing of multi‐functional assembles and enables a polymer‐based diagnosis and treatment platform for liquid biopsy, cell biopsy, and photothermal treatment of ovarian cancer (Figure 1B).

## 2 Results and Discussion

### 2.1 Synthesis and Characterization of OM and SP

**Figure 2:**
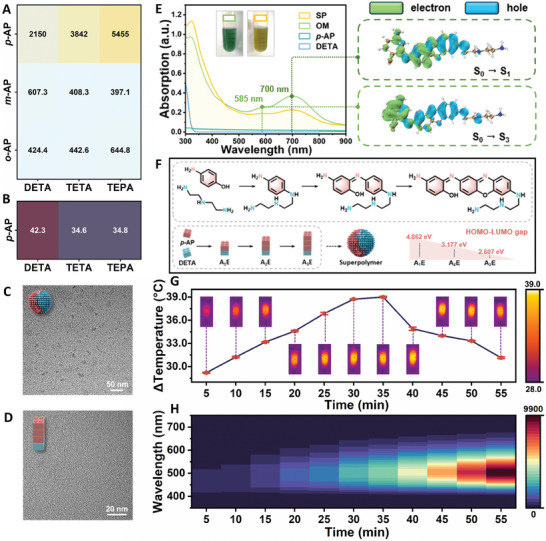
Fluorescent and photothermal properties characterization of OM and SP. Heat map of maximum fluorescence intensity A) and temperature B). TEM results of SP C) and OM D). E) The absorption spectra of DETA, *p*‐AP, OM, and SP. Inset: the image of OM and SP solution, and the hole and electron distribution isosurfaces of S_0_→S_1_ and S_3_ excitation of A_3_E. F) Schematic diagram to illustrate the in‐situ formation of A_1_E, A_2_E, A_3_E, and SP as well as HOMO‐LUMO gap of A_1_E, A_2_E and A_3_E. G) Temperature changes and infrared thermal images of cascade polymer solution with the increase of polymerization time (5–55 min). The polymeric conditions of the cascade polymers:75 µL DETA (20%, *v*/*v*) ethanol solution was mixed with 1 mL *p*‐AP (100 µm) in PIPES buffer (10 mm, pH 7.3). H) Fluorescence intensity of cascade polymers solution with the increase of polymerization time.

The suitable building blocks (monomer and crosslinking agent) for the synthesis of polymers were selected, and the detailed experimental process is described in Supporting Information. As shown in **Figure** **2**A and Figure S1 (Supporting Information), among the selected monomers and crosslinking agents, only *p*‐AP can react with the three crosslinking agents DETA, triethylenetetramine (TETA), and tetraethylenepentamine (TEPA) to produce fluorescent substances, and the fluorescence intensity enhances along with the increase of the *p*‐AP concentration (Figure S2A–C, Supporting Information). Further, the highest occupied molecular orbital (HOMO) and lowest unoccupied molecular orbital (LUMO) energy levels of *o*‐AP, *m*‐AP, and *p*‐AP were obtained by Multiwfn software, and the simulated results are shown in Figure S3 (Supporting Information).^[^ ^40^, ^41^ ^]^ Among the three molecules, *p*‐AP has the highest HOMO, the higher the HOMO energy level, the more conducive it is to lose electrons and the stronger reduction ability, that is, *p*‐AP is more likely to react with a crosslinking agent.^[^ ^42^ ^]^ Additionally, only the reaction of DETA and *p*‐AP can produce an excellent photothermal effect, and the temperature signal is enhanced along with the increase of the *p*‐AP concentration (Figure 2B; Figure S2D, Supporting Information). From Figure S4 (Supporting Information), it can be observed that the fluorescence images under 365 nm UV light and infrared images of the reaction solution reflect the changing trend of fluorescence and temperature. Therefore, *p*‐AP is selected as a monomer, and DETA is selected as crosslinking agent.

Then, the OM and SP were synthesized with *p*‐AP and DETA and characterized. The Fourier transform infrared (FT‐IR) results are shown in Figure S5 (Supporting Information), the peaks of OM and SP near 3426.88 cm^−1^ are attributed to the stretching vibration of ‐NH_2_, and the absorption bands at 1639.20–1317.49/1637.27–1317.14, and 1037.52/1039.44 cm^−1^ of OM and SP belong to C═N, N─H, and C─O/C─N vibration, respectively. As displayed in Figure 2C, SP is nanoparticles with uniform particle size and good dispersion, which is similar to that reported previously.^[^ ^43^ ^]^ At the same time, OM does not form visible nanoparticles in the TEM image (Figure 2D). The powder X‐ray diffraction (PXRD) pattern of SP (Figure S6, Supporting Information) exhibits a broad peak at ≈23°, which is ascribed to the amorphous structure of SP.^[^ ^43^ ^]^ OM was also characterized in the same way, and the result is consistent with that SP, which may be due to oxidation during freeze‐drying. The UV–vis absorption spectra of OM and SP mainly show significant absorption at 300–400 and 500–900 nm (Figure 2E), the colors of OM and SP are dark green and yellow, respectively. As seen in Figure S7 (Supporting Information), the results of X‐ray photoelectron spectroscopy (XPS) demonstrate the existence of C, N, and O in OM and SP, and the high‐resolution spectra of C, N, and O are exhibited. As shown in Figure S7B (Supporting Information), the C 1s spectrum indicates the existence of C─C/C═C (283.73 eV, 283.81 eV), C─N/C─O (284.93 eV, 285.05 eV), and C═N/C═O (287.16 eV, 287.33 eV) bonds in OM and SP. N 1s spectrum shows C═N (398.16 eV, 398.12 eV) and N─H/C─N (400.11 eV, 400.06 eV) bonds in OM and SP (Figure S7C, Supporting Information). As shown in Figure S7D (Supporting Information), the O 1s spectrum shows C═O (529.72 eV, 529.69 eV) and C─O (531.18 eV, 531.15 eV) bonds in OM and SP. The structural results of OM and SP characterized by XPS are consistent with those by FT‐IR.

### 2.2 Fluorescent and Photothermal Properties of OM and SP

During the experiment, we found that there is a conversion process from OM to SP in the solution. For qualitative analysis of OM and SP, mass spectrometry was utilized to determine their compositions. As shown in Figures S8 and S9 (Supporting Information), the mass spectra results indicate that the main components are the same in OM and SP. According to the results of molecular weight and characterization, we inferred the structural formula and cascade polymerization process of three molecules (A_1_E, A_2_E, and A_3_E) as illustrated in Figure 2F. According to previous reports, due to the existence of the imine‐based double‐bond, photochemical rotation around the C═N bond (C═N rotation) and thermal nitrogen‐inversion (N‐inversion) in the molecule make it exhibit the characteristics of “molecular motor” under irradiation.^[^ ^44^ ^]^ Therefore, A_2_E and A_3_E are most likely molecules with photothermal effects due to the active intramolecular motions (rotation, vibration, twisting, etc.). To verify our hypothesis, the electronic absorption spectra of the molecules were calculated by Multiwfn.^[^ ^45^ ^]^ The results show that there is no absorption peak after 300 nm for A_1_E, the absorption peak of A_2_E is ≈450 nm, and the absorption peak of A_3_E is ≈550 and 700 nm (Figure S10, Supporting Information). The superposition of these spectra is very similar to the absorption spectra of cascade polymer solution (Figure 2E), indicating that the molecules we speculated are correct. More importantly, we found that A_3_E is the main contributor to the photothermal effect because of its near‐infrared absorption and small HOMO‐LUMO gap (2.607 eV, facilitating charge transfer) (Figure 2F), while A_1_E, A_2_E do not possess this property.^[^ ^36^ ^]^ To investigate the photothermal effect of A_3_E in detail, we first empirically defined the different parts of A_3_E as donor and acceptor (Figure S11, Supporting Information), and then plotted the charge‐transfer spectrum of A_3_E to visually analyze the internal characteristics of the electronic spectrum.^[^ ^46^ ^]^ As shown in Figure 2E, Figure S10D, and Table S1 (Supporting Information), the calculation results reveal that absorption of A_3_E is attributed to twisted intramolecular charge transfer (TICT) property (A_3_E transition from S_0_ state to S_1_ and S_3_ states), and the hole and electron distribution isosurfaces of A_3_E indicate that the continuous flow of electrons from the donor to the acceptor causes an absorption peak of ≈700 nm. Thus, the photothermal property of OM can be ascribed to the TICT effect and the light‐driven imine‐based intramolecular motion‐enhanced nonradiative pathway.^[^ ^46^, ^47^ ^]^ Furthermore, we proposed the hypothesis on the formation of fluorescent polymer that SP with high fluorescence intensity is formed by OM aggregation. The TEM results support this hypothesis, and aggregates are formed with prolonged polymerization time (Figure 2C,D). In this process, the active intramolecular movement is restricted by aggregation, and the enhancement of the radiative transition pathway leads to the weakening of the photothermal effect and the enhancement of fluorescence (Figure 2G,H).^[^ ^48^, ^49^ ^]^

For the OM with photothermal property, the temperature of the as‐prepared OM solution increases obviously with the prolongation of 808 nm laser irradiation time, while the temperature of *p*‐AP and DETA increases slightly (Figure S12A, Supporting Information). The results show that the OM solution has an excellent NIR laser‐driven photothermal effect. As seen in the UV–vis–NIR absorption spectra of the three solutions (Figure 2E), the OM solution possesses an absorption spreading to the near‐infrared region, which is a necessary condition for realizing near‐infrared photothermal conversion. Additionally, the thermal effect evaluation experiment was carried out to measure the photothermal conversion ability of OM, the photothermal conversion efficiency (*η*) was calculated to be 21.24% (Figure S12B,C, Supporting Information).^[^ ^50^ ^]^ The SP with fluorescence exhibits the maximum emission of 505 nm under the excitation of 380 nm (Figure S13, Supporting Information). As the extension of the reaction time, the fluorescence of the SP solution increases gradually (Figure 2H), while the photothermal effect and absorption in the near‐infrared region increase first and then decrease (Figure 2G; Figure S14, Supporting Information).

### 2.3 Dual‐Mode Assay for β‐Gal in Human Serum

**Figure 3:**
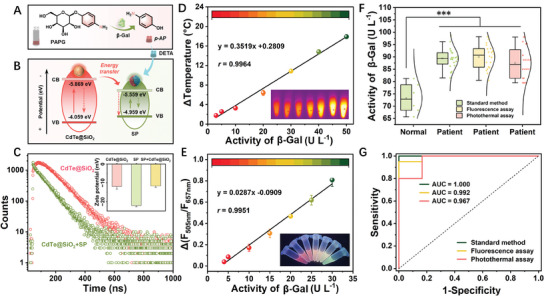
Liquid biopsy analysis of β‐Gal activity based on the photothermal and ratiometric fluorescence dual‐mode method. A) Schematic illustration of PAPG enzymatic hydrolysis by β‐Gal. B) Conduction band and valence band of CdTe@SiO_2_ and SP. C) Decay curves of CdTe@SiO_2_, and CdTe@SiO_2_ + SP. Inset: Zeta potential of SP (1 mg mL^−1^ SP in PIPES buffer (10 mm, pH 7.3)), CdTe@SiO_2_ (200 µL CdTe@SiO_2_ in 2 mL PIPES buffer (10 mm, pH 7.3)), and SP (1 mg mL^−1^ SP in PIPES buffer (10 mm, pH 7.3) + CdTe@SiO_2_ solution (200 µL CdTe@SiO_2_ in 2 mL PIPES buffer (10 mm, pH 7.3)). D) The calibration curve of Δ*T* and β‐Gal activity spiked in the blank serum (3–50 U L^−1^). E) The calibration curve of Δ(*F*_505nm_/*F*_657nm_) and β‐Gal activity spiked in the blank serum (4–30 U L^−1^). Δ(*F*_505nm_/*F*_657nm_) and Δ*T* are the fluorescence ratio difference and the temperature difference, respectively, between the experimental group (with β‐Gal) and blank group (without β‐Gal). Error bars represent the standard deviations of three repetitive experiments. F) The activity of β‐Gal in real serum samples detected by dual‐mode assay and colorimetric assay (standard method), respectively (^***^ *p* < 0.001). G) The receiver operating characteristic (ROC) curves of the standard method, fluorescence, and photothermal assay for the detection of β‐Gal in 20 real serum samples of ovarian cancer patients.

The above results make the fluorescent‐photothermal dual‐mode detection method based on cascade polymers for *p*‐AP‐related targets assay possible. It is worth noting that *p*‐AP can be obtained by β‐Gal hydrolysis of 4‐aminophenyl‐β‐D‐galactopyranoside (PAPG) (**Figure** **3**A), and the feasibility of β‐Gal enzymatic hydrolysis of PAPG is verified (Figure S23, Supporting Information). Therefore, PAPG can be used as a substrate to construct the detection pathway of β‐Gal activity. The design strategy of the β‐Gal assay system is shown in Figure 1B. In the presence of β‐Gal, PAPG is hydrolyzed to *p*‐AP, and *p*‐AP reacts with DETA to produce cascade polymers, which possess photothermal properties and strong fluorescence emission. As seen in Figure S24A (Supporting Information), the reaction system with β‐Gal exhibits the activation of fluorescence and photothermal signals, and other substances (PAPG, DETA, and β‐Gal) do not interfere with dual‐mode signals. In addition, CdTe@SiO_2_ was introduced into the detection system as the fluorescent internal standard and the second fluorescence sensing center, and the synthesis details and characterization results are shown in Supporting Information. As shown in Figure S24B (Supporting Information), fluorescence at 657 nm of CdTe@SiO_2_ is quenched after the polymerizing reaction, and there is a large chromaticity shift between the red fluorescence of CdTe@SiO_2_ and the green fluorescence of the produced SP, which makes it possible to visually detect β‐Gal activity.^[^ ^51^, ^52^ ^]^ Additionally, the conduction band of SP (−5.559 eV) is lower than that of CdTe@SiO_2_ (−5.869 eV), indicating that energy can transfer from CdTe@SiO_2_ to SP (Figure 3B), resulting in CdTe@SiO_2_ fluorescence quenching.^[^ ^53^, ^54^, ^55^, ^56^ ^]^ Through the analysis of Zeta potential and fluorescence lifetime as shown in Figure 3C, it is inferred that there is an electrostatic interaction and dynamic quenching mechanism between SP and CdTe@SiO_2_ (the analysis details in Supporting Information).^[^ ^57^ ^]^

Several parameters that affect the detection performance were optimized, and the optimization details and results are presented in Figures S26 and S27 (Supporting Information). As shown in Figure S28A (Supporting Information), *F*_505nm_ increases gradually and *F*_657nm_ decreases with β‐Gal activity from 0 to 30 U L^−1^. Δ(*F*_505nm_/*F*_657nm_) shows a good linear relationship with β‐Gal activity in the range of 2–30 U L^−1^ (*r* = 0.9971) (Figure S28B, Supporting Information). The limit of detection (LOD) is 0.5 U L^−1^ via the 3σ method (σ is the standard deviation of *F*_505nm_/*F*_657nm_ of eleven blank samples). Meanwhile, the performance of the photothermal assay was also investigated. As seen in Figure S28C (Supporting Information), the thermal response of the solution system progressively increases after irradiation, and Δ*T* increases with the increase of β‐Gal activity. Δ*T* shows a good linear relationship with β‐Gal activity in the range of 3–50 U L^−1^ (*r* = 0.9951) (Figure S28D, Supporting Information), and LOD is 1 U L^−1^ (3σ method, σ is the standard deviation of the temperature of eleven blank samples).

Furthermore, the selectivity and anti‐interference ability of the dual‐mode method were investigated. The common interferences in biological samples were selected, including cations and anions (Na^+^, K^+^, CO_3_^2−^, SO_4_^2−^, CH_3_COO^−^, Br^−^), small molecule substances (glucose, proline, glutamic acid, tyrosine), several enzymes (chymotrypsin, trypsin, lysozyme, alkaline phosphatase, ribonuclease, pepsase), and reductive substances (cysteine, glutathione, ascorbic acid). As shown in Figure S29A–D (Supporting Information), encouragingly, interferents have no significant effect on the dual‐mode detection method, indicating the fluorescent‐photothermal dual‐mode detection strategy based on cascade polymers has satisfactory selectivity and anti‐interference ability in the detection of β‐Gal activity in complex biological samples.

The photothermal and ratiometric fluorescence dual‐mode assay method was applied to detect β‐Gal activity in human serum. The specific assay procedure is shown in Figure S30 (Supporting Information). For the photothermal assay, there is a good linear relationship between Δ*T* and β‐Gal activity in a broad range (3–50 U L^−1^) (Figure 3D), and the equation is y = 0.3519x + 0.2809 (*r* = 0.9964), and LOD is calculated to be 1.5 U L^−1^. For the fluorescence assay, the calibration curve of ratiometric fluorescence change (Δ(*F*_505nm_/*F*_657nm_)) and β‐Gal activity in the range of 4–30 U L^−1^ is constructed, as shown in Figure 3E, the linear equation is y = 0.0287x – 0.0909 (*r* = 0.9951), and the LOD is 1.2 U L^−1^. Serum samples with different β‐Gal activities (low, middle, and high levels) were prepared by the standard addition method. As seen in Table S2 (Supporting Information), the recovery ranges from 87.9% to 109.0% with a relative standard deviation (RSD) of less than 6.4% for the ratiometric fluorescence assay. The photothermal assay provides a recovery of 85.8–101.4% (RSD < 8.6%). These detection results indicate the dual‐mode method has sufficient accuracy and precision for monitoring β‐Gal activity in real serum samples. Furthermore, the ratiometric fluorescence and photothermal dual‐mode assay were applied to β‐Gal activity determination in human serum samples from twenty ovarian cancer patients and six healthy adults. These serum samples were also detected by the commercial kit standard method (colorimetric assay), and the β‐Gal level in the cancer group is generally higher than that in the normal group (^***^ *p* < 0.001) (Figure 3F). The results indicate that the overexpression of β‐Gal in serum is related to ovarian cancer, and it is significant to develop a quantitative detection method of β‐Gal. The β‐Gal level in serum samples of cancer patients is shown in Figure S31 (Supporting Information), and the fluorescence and photothermal assays show 99.2% and 96.7% agreement relative to the standard method, respectively (Figure 3G). Therefore, the dual‐mode assay established in this work is as reliable as the commercial kit for β‐Gal detection.

### 2.4 Fluorescence Imaging of Ovarian Cancer Cells

The application potential of enzyme‐activatable SP for fluorescence imaging of ovarian cancer cells was assessed by confocal fluorescent microscopy. SKOV‐3 cells from human ovarian cancer patients were selected for further experiments because the overexpression of β‐Gal in SKOV‐3 cells has been reported.^[^ ^58^ ^]^ Initially, the cell counting kit‐8 (CCK‐8) assay was utilized to test the cytotoxicity of the substrate (PAPG) and DETA so as to determine their concentration used in cell fluorescence imaging. As shown in Figure S32A–D (Supporting Information), PAPG does not show significant toxicity to normal cells (Vero cells and MRC‐5 cells), only DETA shows mild cytotoxicity to MRC‐5 cells at the maximum concentration.

**Figure 4:**
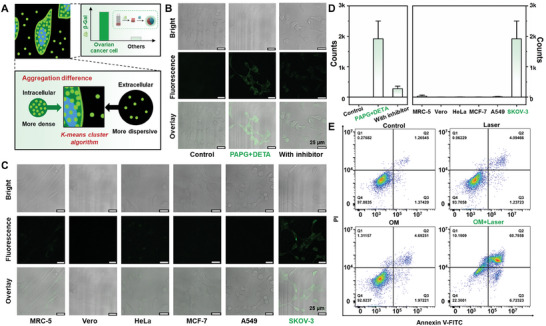
In vitro analysis results of self‐assembled polymer. A) The schematic illustration of the aggregation characteristics of non‐black pixels inside and outside the cell. B) Confocal fluorescence images, bright field images, and overlay images of SKOV‐3 cells after various treatments: without any treatment (first column, control), incubated with PAPG and DETA, respectively (second column), pretreated with D‐galactose (1 mm, inhibitor) for 1 h and then incubated with PAPG and DETA, respectively (third column). Scale bar: 25 µm. C) Confocal fluorescence images, bright field images, and overlay images of MRC‐5, Vero, HeLa, MCF‐7, A549, and SKOV‐3 cells incubated with PAPG and DETA respectively. Scale bar: 25 µm. D) Fluorescence intensity of confocal fluorescence images in B) and C). E) Flow cytometry analysis of cell apoptosis of SKOV‐3 cells treated with control (without any treatment), laser irradiation, OM, OM + laser irradiation. Q1, Q2, Q3, and Q4 quadrants represent necrotic cells, late apoptotic cells, early apoptotic cells, and live cells, respectively, and the number of cells collected in each group is 10 000.

Furthermore, we explored the toxicity of the superimposed PAPG and DETA on cells, and the results are shown in Figure S32E,F (Supporting Information). The combination of PAPG and DETA does not show significant toxicity to Vero cells, and the survival rate of MRC‐5 cells reaches more than 90% when PAPG and DETA are less than 2 mg mL^−1^ and 0.32‰ (*v*/*v*), respectively. As presented in Figure S33 (Supporting Information), the fluorescence images of Vero cells and MRC‐5 cells co‐stained with Calcein AM (green fluorescence, live cells) and propidium iodide (PI) (red fluorescence, dead cells) show no significant cytotoxicity with the treatment of PAPG (2 mg mL^−1^) and DETA (0.32‰ (*v*/*v*)), which is consistent with the results of CCK‐8 analysis. Further, the fluorescence imaging performance of β‐Gal‐activated self‐assembled SP in cells was explored. **Figure** **4**A is the schematic illustration of the aggregation characteristics of non‐black pixels inside and outside the cell. Based on the degree of aggregation, the fluorescence intensity of in‐situ generated SP in cells can be quantitatively analyzed by the K‐means clustering algorithm.^[^ ^59^, ^60^ ^]^ As shown in Figure 4B, there is no green fluorescence in the control cells. In contrast, the experimental group shows obvious green fluorescence in the cytoplasm region after PAPG and DETA treatment, demonstrating that PAPG can permeate into SKOV‐3 cells and be hydrolyzed to *p*‐AP by endogenous β‐Gal, thus triggering the generation of fluorescent SP. When pretreated with a β‐Gal inhibitor (D‐galactose) and then treated with PAPG and DETA, only weak green fluorescence can be observed in SKOV‐3 cells. To investigate the specificity of our proposed in‐situ fluorescence imaging method for monitoring β‐Gal activity in SKOV‐3 cells, PAPG and DETA were also incubated with MRC‐5, Vero, HeLa, MCF‐7, and A549 cells. Significantly, the fluorescent signal in SKOV‐3 cells is much brighter than that in negative control cells (Figure 4C). Moreover, due to the high expression of β‐Gal in SKOV‐3 cells and the mechanism of SP formation, SP trends to present high concentration and high aggregation degree in the cell. Therefore, we developed the K‐means clustering algorithm that takes the “fluorescence intensity” and “aggregation degree” as 2D analysis indexes to quantitatively analyze fluorescent confocal images, and the quantitative results (Figure 4D) can distinguish the target cells (SKOV‐3 cells) from other cells more easily compared to the naked‐eye observation (Figure 4B,C), which shows a promising application prospect in clinical analysis.

### 2.5 Photothermal Therapy Efficiency of OM on Ovarian Cancer

To evaluate the photothermal properties of synthetic OM, it was exposed to the NIR laser at different power densities for 3 min (808 nm, power density of 0.8–2.2 W cm^−2^). As shown in Figure S35A (Supporting Information), the temperature of OM under laser irradiation rises rapidly, and the temperature increases with the increase of laser power density. Further, the photostability of synthetic OM was investigated. As shown in Figure S35B (Supporting Information), four cycles of on/off NIR laser irradiation were performed on OM, and the temperature change of OM basically remains consistent. It illustrates that OM has good photothermal performance, which can ensure its therapeutic effect as a photothermal agent. Next, the thermal effect evaluation experiment was carried out to measure the photothermal conversion ability of the synthetic OM and indocyanine green (ICG) (contrast photothermal agent, 20 µm), the *η* was calculated to be 43.0% and 40.8%, respectively (Figure S36, Supporting Information). To ensure the biosafety of OM, the CCK‐8 assay and confocal imaging experiments with Calcein‐AM and PI cell staining were performed to evaluate the cytotoxic effect of OM on Vero cells. As shown in Figure S37 (Supporting Information), different volumes of OM and 808 nm laser irradiation alone have no significant effect on the survival rate of the cells, and the results of the cell staining experiments (Figure S38, Supporting Information) are consistent with those of the CCK‐8 assay, indicating that OM has good biosafety. Ovarian cancer cell SKOV‐3 was used to investigate the therapeutic effect of OM. The CCK‐8 assay results are shown in Figure S39 (Supporting Information), after incubation with different volumes of OM for 12 h, cells still maintain a viability of over 80%. However, the survival rate of cells treated with OM plus 808 nm laser irradiation is much lower and decreases with the increase of OM volume, the cell viability is only 26% after incubation with 10 µL OM. The results show that OM has weak toxicity to SKOV‐3 cells without laser irradiation but can produce a significant killing effect on the cells after laser irradiation, and the results of the cell staining assay (Figure S40, Supporting Information) are consistent with those of the CCK‐8 assay. The apoptosis of SKOV‐3 cells was also quantitatively studied by flow cytometric analysis. As shown in Figure 4E and Figure S41 (Supporting Information), the apoptosis rate in the photothermal treatment group is 67.5%, while the apoptosis rate in other groups is lower than 10%. The above experimental results indicate that OM can effectively kill SKOV‐3 cells under laser irradiation and potentially be used as photothermal agents in photothermal therapy (PTT).

**Figure 5:**
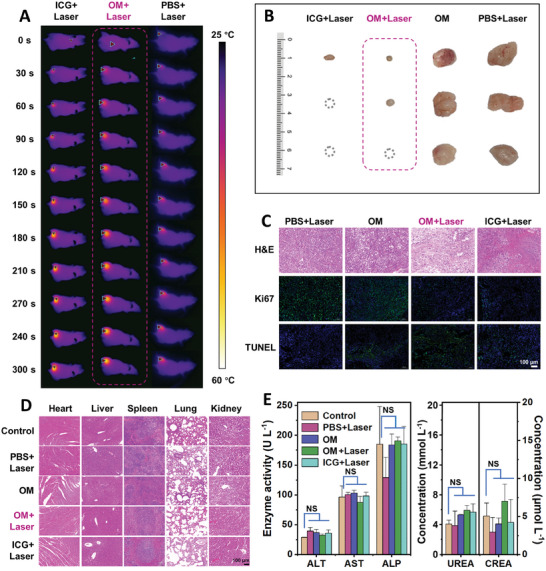
In vivo evaluation of anti‐tumor effects of self‐assembled polymer in SKOV‐3 primary tumor‐bearing mouse models. A) Representative infrared photographs of SKOV‐3 tumor‐bearing model treated with ICG, OM, and PBS solution with 808 nm laser irradiation (1.0 W cm^−2^). B) Tumor images of mice after different treatments for 15 days. C) H&E, Ki67, and TUNEL stained histological images of tumor slices collected from different groups of mice after treatment for 24 h (scale bar: 100 µm). D) H&E staining of major organs (heart, liver, spleen, lung, and kidney) of normal nude mice (control) and tumor‐bearing nude mice represented by each group at the end of the 15‐day course. E) Biochemical analysis results of normal nude mice (control) and tumor‐bearing nude mice in each group, including alanine aminotransferase (ALT), aspartate aminotransferase (AST), alkaline phosphatase (ALP), urea, and creatinine (CREA) (NS: no significant, *p* > 0.05).

Based on the in vitro experimental results, OM has excellent photothermal conversion capabilities and the potential to be used in the PTT of tumors. Therefore, we first established a SKOV‐3 subcutaneous tumor model to investigate the photothermal imaging effects of OM (synthesized with *p*‐AP and DETA in vitro) and selected the photothermal agent ICG as a control group. When the tumor volume reached ≈110 mm^3^, phosphate buffer solution (PBS), OM, and ICG were intratumorally injected, respectively. The tumor site was irradiated with 808 nm laser (1.0 W cm^−2^) for 5 min, and the temperature changes were recorded with the infrared imaging device. As seen in **Figure** **5**A and Figure S42A (Supporting Information), it can be intuitively seen that the temperature at the tumor site in the PBS + Laser group increases by ≈9 °C, which is not enough to kill tumor cells. In the OM + Laser and ICG + Laser groups, the temperature at the tumor site is significantly increased by ≈20 °C, demonstrating that OM can produce excellent photothermal effects, and also has the ability to induce tumor cell apoptosis.

Next, we further studied the PTT efficiency of OM on mouse tumors. The tumor‐bearing mice were randomly divided into four groups: a) PBS + Laser group, b) OM injection group, c) OM + Laser group, d) ICG + Laser group. And corresponding treatments were given to evaluate the anti‐tumor effect of OM in vivo. The treatment results are shown in Figure 5B, Figures S42B and S43 (Supporting Information). It shows that the growth of tumors in the OM + Laser group and the ICG + Laser group is effectively inhibited, and even tumor ablation is achieved. Fifteen days later, the mice are sacrificed, and it can also be seen that the tumor growth rate in the photothermal treatment group (OM + Laser group and ICG + Laser group) is significantly slower than that in the PBS + Laser group and OM group (^**^ *p *< 0.01) (Figure S42C, Supporting Information), indicating that the effect of PTT with OM as photothermal agents is comparable to that of ICG. However, only laser irradiation or OM injection cannot achieve the effect of tumor treatment. Additionally, hematoxylin and eosin (H&E) staining, TdT‐mediated dUTP nick end labeling (TUNEL) staining, and Ki67 immunofluorescence staining were performed on tumor tissue after PTT to analyze cell status, apoptosis, and proliferation (Figure 5C). As expected, in the OM + Laser group, tumor cell damage is most significant, and the proliferative ability of tumor cells becomes weaker. In addition, the biosafety of OM was also evaluated. As shown in Figure S42D (Supporting Information), there is no significant change in the body weight of mice in the OM + Laser group during treatment, indicating that the OM is nontoxic to the mice. Finally, the major organs (heart, liver, spleen, lung, and kidney) were stained with H&E for histopathological analysis. The H&E staining results show that the histological morphology of the organs of treated mice is consistent with that of completely healthy mice (Figure 5D; Figure S44, Supporting Information), further illustrating the biosafety of OM for tumor treatment. The analysis results of biochemistry indexes including alanine aminotransferase (ALT), aspartate aminotransferase (AST), alkaline phosphatase (ALP), urea, and creatinine (CREA) in each group are within the normal range, and there is no significant difference compared to the control group (Figure 5E).

Acute toxicity studies are important to evaluate the main effects of compounds. In the acute toxicity study, OM (50, 100, 150, and 200 µL) does not cause any mortality or signs of toxicity in nude mice during the experimental period (7 days). Likewise, the animal groups treated with OM do not present significant alterations in body weight during the experimental period compared to the control group, as shown in Figure S45 (Supporting Information), demonstrating that OM cannot affect the normal growth of the animals. Additionally, as shown in Figures S46 and S47 (Supporting Information), the macroscopic characteristics of organs and the H&E staining results indicate that OM does not induce alterations in the organs' tissues. Furthermore, the data from biochemical analysis demonstrate that there is no significant difference between the OM‐treated groups and the control group (Figure S48, Supporting Information). Because the results of the acute toxicity experiment prove that there are no abnormalities and no deaths, even if the administered dose of OM is 200 µL (4 times the therapeutic dose). Therefore, the dose of OM administered to mice is set at 200 µL in the acute toxicity experiment, which is also used as the maximum tolerated dose in mice.

The comet assay was employed to determine the DNA damage induced by OM with MRC‐5 cells as a model. Compared to the PBS group (negative control, Figure S49A, Supporting Information), the methyl methanesulfonate group (positive control, Figure S49B, Supporting Information) shows significant DNA damage, as there are a lot of damaged cells with tails longer than the nucleus diameter. On the other hand, the 40 µL of OM exhibits similar results to the PBS group, indicating it does not promote DNA damage (Figure S49C, Supporting Information). However, the 60 µL of OM is able to promote weak DNA damage because the cells show a slight tailing (Figure S49D, Supporting Information). These results suggest that only the OM in high doses is genotoxic. In summary, the above research results demonstrate that OM has good biosafety and excellent photothermal performance, and has high PTT efficiency for ovarian cancer treatment.

## 3 Conclusion

This study reports a hierarchical self‐driven polymer with photothermal and fluorescent properties, and the mechanism of multi‐step polymerization similar to the assembly of building blocks has been reasonably proposed. The intracellular expression of the enzyme activation pathway is an effective strategy for selective activation of self‐assembly. β‐Gal was used as the activation model enzyme to effectively verify its ability to trigger the hierarchical polymer self‐assembly, and a sensitive dual‐mode assay method of β‐Gal activity was constructed for the detection of β‐Gal in the serum samples of ovarian cancer patients. In vitro fluorescence imaging visualization analysis was realized by using the polymer selective self‐assembly in ovarian cancer cells. The photothermal property of the polymer has also been validated in animal trials in subcutaneous tumor models, achieving satisfactory therapeutic effects on ovarian cancer. Overall, this work pioneers self‐driven cascade polymers with fluorescent and photothermal properties, introducing a profound dimension for the construction of advanced intelligent materials. The combination of the diversity of polymeric building blocks and the designability of enzyme substrates can meet the needs of enzyme activation strategies in more types of cancer cells, expanding the application of polymers in the diagnosis and treatment of more diseases.

## 4 Experimental Section

Experimental details are provided in Supporting Information.

## Conflict of Interest

The authors declare no conflict of interest.

## Supporting information

Supporting Information

## Data Availability

The data that support the findings of this study are available from the corresponding author upon reasonable request.

## References

[1] Y. Zhang , J. Wang , Y. He , J. Pan , X. Jin , J. Shang , G. Gong , J. J. Richardson , I. Manners , J. Guo , Angew. Chem., Int. Ed. 2023, 62, e202303463. 10.1002/anie.202303463 37208956

[2] P. Roth , R. Meyer , I. Harley , K. Landfester , I. Lieberwirth , M. Wagner , D. Y. W. Ng , T. Weil , Nat. Synth. 2023, 2, 980.

[3] X. Bai , Q. Sun , H. Cui , L. P. B. Guerzoni , S. Wuttke , F. Kiessling , L. De Laporte , T. Lammers , Y. Shi , Adv. Mater. 2022, 34, 2109701. 10.1002/adma.202109701 35906820

[4] F. Jehle , P. Fratzl , M. J. Harrington , ACS Nano 2018, 12, 2160. 29385330 10.1021/acsnano.7b07905

[5] Z. Jiang , Y. Liu , R. Shi , X. Feng , W. G. Xu , X. Zhuang , J. Ding , X. Chen , Adv. Mater. 2022, 34, 2110094. 10.1002/adma.202110094 35202501

[6] P. Zhang , Y. Liu , G. Feng , C. Li , J. Zhou , C. Du , Y. Bai , S. Hu , T. Huang , G. Wang , P. Quan , J. Hirvonen , J. Fan , H. A. Santos , D. Liu , Adv. Mater. 2023, 35, 2211254. 10.1002/adma.202211254 36802103

[7] F. Xiao , X. Fang , H. Li , H. Xue , Z. Wei , W. Zhang , Y. Zhu , L. Lin , Y. Zhao , C. Wu , L. Tian , Angew. Chem., Int. Ed. 2022, 61, e202115812. 10.1002/anie.202115812 35064628

[8] W. Li , L. Shi , Y. Wu , F. Wei , J. Fu , C. Jing , J. Cheng , S. Liu , Energy Stor. Mater. 2022, 53, 183.

[9] S. Götz , S. Zechel , M. D. Hager , G. R. Newkome , U. S. Schubert , Prog. Polym. Sci. 2021, 119, 1.

[10] H. Zhu , H. Wang , B. Shi , L. Shangguan , W. Tong , G. Yu , Z. Mao , F. Huang , Nat. Commun. 2019, 10, 2412. 31160596 10.1038/s41467-019-10385-9 PMC6546686

[11] S. Gentile , E. Del Grosso , L. J. Prins , F. Ricci , Angew. Chem., Int. Ed. 2021, 60, 12911. 10.1002/anie.202101378 33783934

[12] Y. Bai , Q. Luo , J. Liu , Chem. Soc. Rev. 2016, 45, 2756. 27080059 10.1039/c6cs00004e

[13] L. Yao , K. Fu , X. Wang , M. He , W. Zhang , P. Y. Liu , Y. P. He , G. Liu , ACS Nano 2023, 17, 2159. 36648130 10.1021/acsnano.2c08315

[14] V. Mihali , M. Skowicki , D. Messmer , C. G. Palivan , Nano Today 2023, 48, 101741.

[15] M. Wang , G. Lv , H. An , N. Zhang , H. Wang , Angew. Chem., Int. Ed. 2022, 61, e202113649. 10.1002/anie.202113649 34994999

[16] J. Guo , B. L. Tardy , A. J. Christofferson , Y. Dai , J. J. Richardson , W. Zhu , M. Hu , Y. Ju , J. Cui , R. R. Dagastine , I. Yarovsky , F. Caruso , Nat. Nanotechnol. 2016, 11, 1105. 27723730 10.1038/nnano.2016.172

[17] Z. Chai , A. Childress , A. A. Busnaina , ACS Nano 2022, 16, 17641. 36269234 10.1021/acsnano.2c07910 PMC9706815

[18] P. Khanra , A. K. Singh , L. Roy , A. Das , J. Am. Chem. Soc. 2023, 145, 5270. 36797682 10.1021/jacs.2c12894

[19] M. Delbianco , A. Kononov , A. Poveda , Y. Yu , T. Diercks , J. Jimenez‐Barbero , P. H. Seeberger , J. Am. Chem. Soc. 2018, 140, 5421. 29624385 10.1021/jacs.8b00254

[20] D. Lauzon , A. Vallee‐Belisle , Nat. Chem. 2023, 15, 458. 36759713 10.1038/s41557-022-01127-4

[21] U. A. Matulonis , A. K. Sood , L. Fallowfield , B. E. Howitt , J. Sehouli , B. Y. Karlan , Nat. Rev. Dis. Primers 2016, 2, 16061. 27558151 10.1038/nrdp.2016.61 PMC7290868

[22] Q. Li , Q. Song , Z. Zhao , Y. Lin , Y. Cheng , N. Karin , Y. Luan , ACS Nano 2023, 17, 10376. 37194951 10.1021/acsnano.3c00804

[23] X. Zheng , X. Wang , X. Cheng , Z. Liu , Y. Yin , X. Li , Z. Huang , Z. Wang , W. Guo , F. Ginhoux , Z. Li , Z. Zhang , X. Wang , Nat. Cancer 2023, 4, 1138. 37488416 10.1038/s43018-023-00599-8 PMC10447252

[24] Y. Zhang , Z. Liu , B. D. Thackray , Z. Bao , X. Yin , F. Shi , J. Wu , J. Ye , W. Di , Small 2018, 14, 1801022. 10.1002/smll.201801022 29974621

[25] J. Zhang , X. Yin , C. Li , X. Yin , Q. Xue , L. Ding , J. Ju , J. Ma , Y. Zhu , D. Du , R. L. Reis , Y. Wang , Adv. Mater. 2022, 34, 2110690. 10.1002/adma.202110690 35275432

[26] X. Mu , F. Wu , Y. Tang , R. Wang , Y. Li , K. Li , C. Li , Y. Lu , X. Zhou , Z. Li , Aggregate 2022, 3, 1.

[27] Y. Wang , W. Du , T. Zhang , Y. Zhu , Y. Ni , C. Wang , F. M. Sierra Raya , L. Zou , L. Wang , G. Liang , ACS Nano 2020, 14, 9585. 32806081 10.1021/acsnano.9b10144

[28] H. Zhou , Y. Zhang , R. R. Zhang , M. Zhao , W. Chen , Y. H. Liu , Y. Jiang , Q. Li , Q. Q. Miao , M. Y. Gao , Adv. Mater. 2023, 35, 2211485. 10.1002/adma.202211485 37086426

[29] C. Xu , S. He , X. Wei , J. Huang , M. Xu , K. Pu , Adv. Mater. 2023, 35, 2211651. 10.1002/adma.202211651 37074842

[30] Z. Zhu , Q. Wang , X. Chen , Q. Wang , C. Yan , X. Zhao , W. Zhao , W. Zhu , Adv. Mater. 2022, 34, 2107444. 10.1002/adma.202107444 34693566

[31] X. Zhen , J. Zhang , J. Huang , C. Xie , Q. Miao , K. Pu , Angew. Chem., Int. Ed. 2018, 57, 7804. 10.1002/anie.201803321 29665259

[32] M. Yi , F. Wang , W. Tan , J. T. Hsieh , E. H. Egelman , B. Xu , J. Am. Chem. Soc. 2022, 144, 13055. 35849554 10.1021/jacs.2c05491 PMC9339482

[33] Y. Wen , N. Jing , M. Zhang , F. Huo , Z. Li , C. Yin , Adv. Sci. 2023, 10, 2206681. 10.1002/advs.202206681 PMC10015879 36651112

[34] P. Liang , Y. Zhang , B. F. Schmidt , B. Ballou , W. Qian , Z. Dong , J. Wu , L. Wang , M. P. Bruchez , X. Dong , Small 2023, 19, 2207535. 10.1002/smll.202207535 36807550

[35] Y. Zhao , X. Zhang , Z. Li , S. Huo , K. Zhang , J. Gao , H. Wang , X. Liang , Adv. Mater. 2017, 29, 1601128. 10.1002/adma.201601128 28639395

[36] S. Li , Z. Wei , L. Xiong , Q. Xu , L. Yu , Y. Xiao , Anal. Chem. 2022, 94, 17263. 36463539 10.1021/acs.analchem.2c04218

[37] L. Lu , L. Guo , K. Wang , Y. Liu , M. Xiao , Biotechnol. Adv. 2020, 39, 107465. 31689470 10.1016/j.biotechadv.2019.107465

[38] K. Gu , Y. Xu , H. Li , Z. Guo , S. Zhu , S. Zhu , P. Shi , T. D. James , H. Tian , W. Zhu , J. Am. Chem. Soc. 2016, 138, 5334. 27054782 10.1021/jacs.6b01705

[39] L. Xu , H. Chu , D. Gao , Q. Wu , Y. Sun , Z. Wang , P. Ma , D. Song , Anal. Chem. 2023, 95, 2949. 36695319 10.1021/acs.analchem.2c04705

[40] T. Lu , F. Chen , J. Comput. Chem. 2012, 33, 580. 22162017 10.1002/jcc.22885

[41] Z. Jiang , Y. Zou , T. Zhao , S. Zhen , Y. Li , C. Huang , Angew. Chem., Int. Ed. 2020, 59, 3300. 10.1002/anie.201913748 31825124

[42] H. Yang , J. Zha , P. Zhang , Y. Qin , T. Chen , F. Ye , Sens. Actuators, B 2017, 247, 469.

[43] J. An , Y. Hu , G. Liu , M. Chen , R. Chen , Y. Lyu , M. Yuan , M. Luo , Y. Liu , J. Mater. Chem. B 2021, 9, 2998. 33635306 10.1039/d0tb02531c

[44] J. S. Ni , X. Zhang , G. Yang , T. Kang , X. Lin , M. Zha , Y. Li , L. Wang , K. Li , Angew. Chem., Int. Ed. 2020, 59, 11298. 10.1002/anie.202002516 32285540

[45] Z. Liu , X. Wang , T. Lu , A. Yuan , X. Yan , Carbon 2022, 187, 78.

[46] F. Lv , X. Fan , D. Liu , F. Song , Acta Biomater. 2022, 149, 16. 35817339 10.1016/j.actbio.2022.07.004

[47] J. Wang , J. Li , L. Wang , T. Han , D. Wang , B. Tang , ACS Appl. Polym. Mater. 2020, 2, 4306.

[48] S. Liu , Y. Li , R. T. K. Kwok , J. W. Y. Lam , B. Tang , Chem. Sci. 2020, 12, 3427. 34163616 10.1039/d0sc02911d PMC8179408

[49] L. Yu , H. Chen , J. Yue , X. Chen , M. Sun , H. Tan , A. M. Asiri , K. A. Alamry , X. Wang , S. Wang , Anal. Chem. 2019, 91, 5913. 30986040 10.1021/acs.analchem.9b00319

[50] X. Li , L. Yang , C. Men , Y. Xie , J. Liu , H. Zou , Y. Li , L. Zhan , C. Huang , Anal. Chem. 2019, 91, 4444. 30811173 10.1021/acs.analchem.8b05031

[51] H. Chen , S. Wang , H. Fu , H. Xie , W. Lan , L. Xu , L. Zhang , Y. She , Spectrochim. Acta A Mol. Biomol. Spectrosc. 2020, 234, 118248. 32179466 10.1016/j.saa.2020.118248

[52] Y. Feng , L. Zhong , Y. Hou , S. Jia , J. Cui , J. Biotechnol. 2019, 306, 54. 31550490 10.1016/j.jbiotec.2019.09.010

[53] Y. Wang , Z. Xie , G. Gotesman , L. Wang , B. P. Bloom , T. Z. Markus , D. Oron , R. Naaman , D. H. Waldeck , J. Phys. Chem. C 2012, 116, 17464.

[54] J. N. Hao , Y. Li , Adv. Funct. Mater. 2019, 29, 1903058.

[55] C. L. Anderson , N. Dai , S. J. Teat , B. He , S. Wang , Y. Liu , Angew. Chem., Int. Ed. 2019, 58, 17978. 10.1002/anie.201908609 31589803

[56] T. Sun , R. Fan , R. Xiao , T. Xing , M. Qin , Y. Liu , S. Hao , W. Chen , Y. Yang , J. Mater. Chem. A 2020, 8, 5587.

[57] A. Karpe , A. Parab , G. Ganesan , P. Walke , A. Chaskar , J. Photochem. Photobiol. A 2022, 431, 114004.

[58] J. Zhang , P. Cheng , K. Pu , Bioconjug. Chem. 2019, 30, 2089. 31269795 10.1021/acs.bioconjchem.9b00391

[59] D. Grün , A. Lyubimova , L. Kester , K. Wiebrands , O. Basak , N. Sasaki , H. Clevers , Nature 2015, 525, 251. 26287467 10.1038/nature14966

[60] V. Y. Kiselev , K. Kirschner , M. T. Schaub , T. Andrews , A. Yiu , T. Chandra , K. N. Natarajan , W. Reik , M. Barahona , A. R. Green , M. Hemberg , Nat. Methods 2017, 14, 483. 28346451 10.1038/nmeth.4236 PMC5410170

