# Supplementary material for: Hierarchical Self‐Assembly Molecular Building Blocks as Intelligent Nanoplatforms for Ovarian Cancer Theranostics
Source: Adv Sci (Weinh). 2024 Feb 26;11(17):2309547. doi: 10.1002/advs.202309547 (PMC11077652; doi:10.1002/advs.202309547)
Supplement: Supplementary file 1 — Supporting Information [file ADVS-11-2309547-s001.pdf]

ADVANCED SCIENCE
Open Access

Supporting Information

for *Adv. Sci.*, DOI 10.1002/advs.202309547

Hierarchical Self-Assembly Molecular Building Blocks as Intelligent Nanoplatforms for Ovarian Cancer Theranostics

Shuo Li, Qingrong Chen, Qi Xu, Zhongyu Wei, Yongjin Shen, Hua Wang, Hongbing Cai, Meijia Gu* and Yuxiu Xiao**

Supporting Information

Hierarchical Self-Assembly Molecular Building Blocks as Intelligent Nanoplatforms for Ovarian Cancer Theranostics

Shuo Li,[1,2] Qingrong Chen,[1] Qi Xu,[1] Zhongyu Wei,[1] Yongjin Shen,[1] Hua Wang,[3] Hongbing Cai,[3,*] Meijia Gu,[1,*] Yuxiu Xiao[1,*]

[1] Department of Thyroid and Breast Surgery, Zhongnan Hospital of Wuhan University, Key Laboratory of Combinatorial Biosynthesis and Drug Discovery (Ministry of Education), School of Pharmaceutical Sciences, Wuhan University, Wuhan, 430071, China

[2] Jiangsu Institute of Hematology, National Clinical Research Center for Hematologic Diseases, NHC Key Laboratory of Thrombosis and Hemostasis, The First Affiliated Hospital and Collaborative Innovation Center of Hematology, Soochow University, Suzhou, 215006, China

[3] Department of Gynecological Oncology, Zhongnan Hospital of Wuhan University, Hubei Key Laboratory of Tumor Biological Behaviors, Hubei Cancer Clinical Study Center, Wuhan, 430071, China

* Corresponding authors.

E-mail address: ZN000738@whu.edu.cn (H. C.); mjgu@whu.edu.cn (M. G.); yuxiuxiao2011@whu.edu.cn (Y. X.).

Experimental

Reagents and Instruments

4-Aminophenyl-β-D-Galactopyranoside (PAPG), tetraethyl orthosilicate (TEOS), $Cd(NO_3)_2·4H_2O$, glutathione, and ascorbic acid were purchased from Aladdin Chemistry Co., Ltd. (Shanghai, China). β-Galactosidase (β-Gal), alkaline phosphatase, ribonuclease, chymotrypsin, trypsin, lysozyme, and pepsase were purchased from Sigma-Aldrich Chemical Reagent Co., Ltd. (Shanghai, China). Glucose was obtained from Shiyi Chemical Reagent Co., Ltd. (Shanghai, China). Glutamic acid was provided by Yuanhang Chemical Co., Ltd. (Tianjin, China). Cysteine was purchased from Ruji Biotechnology Co., Ltd. (Shanghai, China). N-acetyl-L-cysteine (NAC) was obtained from Yuanye Chemical Co., Ltd. (Shanghai, China). Cetyltrimethylammonium bromide (CTAB), $NaBH_4$, ethanol, HCl, NaOH, KCl, NaCl, KBr, CH_3COONa, Na_2CO_3, and Na_2SO_4 were acquired from Sinopharm Chemical Reagent Co., Ltd. (Shanghai, China). Diethylenetriamine (DETA), triethylenetetramine (TETA), tetraethylenepentamine (TEPA), *o*-aminophenol (*o*-AP), *m*-aminophenol (*m*-AP), *p*-aminophenol (*p*-AP), Na_2TeO_3, methyl methanesulfonate (MMS), and 1,4-piperazinediethanesulfonic acid (PIPES) were obtained from Macklin Biochemical Co., Ltd. (Shanghai, China). β-Galactosidase kit was purchased from Elabscience Biotechnology Co., Ltd. (Wuhan, China). Dulbecco's modified Eagle's media (DMEM) medium and McCoy's 5A medium were purchased from Gibco. Phosphate buffered saline (PBS), penicillin and streptomycin were purchased from Invitrogen. Fetal bovine serum (FBS) and trypsin-EDTA were obtained from Thermo Fisher Scientific. Cell counting kit-8 was purchased

from yeasen Biotechnology Co., Ltd. (Shanghai, China). Live/dead cell double staining kit was purchased from Bioscience Technology Co., Ltd. (Shanghai, China). Deionized water (18 Ω·cm) was obtained from a Milli-Q purification system (Millipore, Molsheim, France). The β-Gal solution was prepared with 10 mM PIPES buffer (pH 7.3) as solvent, and all the other solutions were prepared with water as solvent.

Powder X-ray diffraction (PXRD) pattern was obtained on a D8 Advance diffractometer (Bruker AXS, Germany) to characterize the structure of polymers. A Fourier transform infrared (FT-IR) spectrometer (PerkinElmer, USA) was used to obtain the FT-IR spectra. The morphology and size of polymers and quantum dots (QDs) were observed by JEM-2100 transmission electron microscopy (TEM, JEOL, Japan). The elemental analysis of polymers and QDs was performed on K-Alpha X-ray photoelectron spectroscopy (XPS, Thermo, USA). The excitation and emission spectra were tested on an FL-4700 fluorescence spectrometer (Hitachi, Japan), and the ultraviolet spectrum was measured by a UV-2600 ultraviolet spectrometer (Shimadzu, Japan). Constant temperature oscillator (MTC-100, Hangzhou Min Instruments Co., Ltd., China) was used to incubate samples. Zeta potential and hydrodynamic diameter were obtained by ZSP Nano Zetasizer (Malvern, UK). The absolute quantum efficiency and decay curves were measured using an Edinburg FLS980 fluorescence spectrometer (Edinburg, UK). The mass spectra were obtained by Q Exactive ultra-high resolution mass spectrometer (Thermo, USA). The 808 nm near-infrared laser (HW808AD) was purchased from Shenzhen Infrared Laser Techn. Co., Ltd (China). The infrared images were taken by MobIR Air portable infrared cameras (GuideIR, China). The absorbance

of the samples was measured using Tecan Spark microplate reader (Tecan, Switzerland). Fluorescence images were collected on Leica-LCS-SP8-STED confocal laser scanning microscope (Leica, Germany) and fluorescence microscope (Olympus, Japan).

Selection of Monomer and Crosslinking Agent

o-AP, *m*-AP, and *p*-AP were selected as candidates for monomers. 40 μL monomer (5 mM) and different volume of DETA (20%, *v*/*v*) ethanol solution were added in 960 μL PIPES buffer (10 mM, pH 7.3), reacting for 30 min at room temperature. Additionally, DETA, TETA, and TEPA were selected as candidates for crosslinking agent. 50 μL of crosslinking agent (20%, *v*/*v*) ethanol solution was added into different concentrations of *p*-AP (0–200 μM), reacting for 30 min at room temperature. For the photothermal assay, the resulting solution was irradiated for 3 min by taking advantage of 808 nm laser (1.5 W cm^{-2}). After irradiation, the temperature of the solution was recorded by using smartphone-assisted portable infrared imager. For fluorescence assay, the solution was transferred to a cuvette to scan the fluorescence spectrum (λ_{ex} = 380 nm).

Preparation of OM and SP for Characterization

For the preparation of OM, 40 μL *p*-AP (5 mM) was added in 960 μL PIPES buffer (10 mM, pH 7.3), and then 75 μL of DETA (20%, v/v) ethanol solution was added, reacting for 30 min at room temperature. For the preparation of SP, the solution was prepared by the above method and reacted for 50 min at room temperature. Then, the OM and SP solutions were taken for TEM test and mass spectrometry, respectively. Moreover,

the OM and SP solution were separately transferred into a dialysis bag (500 Da) to separate from the reactants and freeze-dried overnight to obtain a polymer solid, which was used for Powder X-ray diffraction (PXRD), FT-IR spectrum analysis and X-ray photoelectron spectroscopy (XPS) analysis.

Calculation of Photothermal Conversion Efficiency (η)

The photothermal conversion efficiency (η) was calculated according to the Eq. 1

$$\eta = \frac{mc(T_{\max} - T_{\max,H_2O})}{I(1-10^{-A})\tau_s} \qquad (1)$$

wherein A is the absorbance of the OM at 808 nm, the value of A is 0.13; m is the mass of OM, herein the m of is 0.2 g; c equals to 4.2 J/g·K, which is the heat capacity of water; I is the power of laser, being set as 1.5 W cm^{-2}; $T_{\max,H_2O}$ and $T_{\max}$ are the maximum temperature values of H_2O and reaction system, which are 33.9°C and 54.3°C, respectively; τ_s is a constant number for a given material, and it was calculated according to the Eq. 2

$$t = -\tau_s \cdot ln\left(\frac{T - T_{surr}}{T_{max} - T_{surr}}\right) = -\tau_s \cdot ln(\theta) \qquad (2)$$

In which t is the time that responds to the realtime change of the reaction system temperature (T), T_{surr} is the temperature of the surrounding and equals to 26.0°C. Then, τ_s is calculated as 207.91 s according to the linear regression of the cooling profile (Figure S12B,C).

Preparation of CdTe and CdTe@SiO_2

The CdTe quantum dots (QDs) and CdTe@SiO_2 were prepared by a hydrothermal

method according to the previous studies. $Cd(NO_3)_2·4H_2O$ (77.1 mg, 0.25 mM) and N-acetyl-L-cysteine (49 mg, 0.3 mM) were dissolved in 18 mL deionized water, and the pH of the mixture was adjusted to 9 with 1 M of NaOH aqueous solution under vigorous stirring. The cadmium precursor solution was then added into the Teflon-lined stainless steel reactor and stirred under nitrogen atmosphere for 10 min. Under nitrogen atmosphere, 1 mL $NaBH_4$ (5.7 mg mL^{-1}) and 1 mL Na_2TeO_3 (10.8 mg mL^{-1}) aqueous solution were injected into the reactor respectively and reacted at 200°C for 50 min. After the reaction, the obtained CdTe QDs were centrifugated at 10,000 rpm for 5 min to remove the solid impurities.

For the synthesis of CdTe@SiO_2, 50 μL CTAB (0.5 mg mL^{-1}) aqueous solution and different volumes of TEOS (2 μL, 4 μL, 6 μL, 8 μL, and 10 μL) were added into 2 mL CdTe QDs. The mixture solution was stirred for 8 h at room temperature. The obtained CdTe@SiO_2 QDs were centrifugated at 10,000 rpm for 5 min to remove the solid impurities, and stored in 4°C before use.

Theoretical Calculation

Theoretical calculations were performed via Gaussian 09 package. The frontier molecular orbital energy of molecules was calculated by the density functional theory (DFT) method, and the polarization function was set at B3LYP/6-31G+ level. The frontier molecular orbital energy results of molecules were exported for graphic analysis by Multiwfn program and visual molecular dynamics (VMD) software.

Cyclic Voltammetry Measurement

A conventional three-electrode cell setup was used with $Ag/AgNO_3$ as the reference electrode, a Pt wire as the counter electrode and a glassy carbon electrode (GCE, 4 mm in diameter) as the working electrode. The supporting electrolyte was a 100 mM acetonitrile solution of tetrabutylammoniumhexafluorophosphate ($TBAPF_6$). The scan rate was set as 50 $mV \cdot s^{-1}$ and the potential range was set between 0 and 1.2 V. The CdTe@SiO_2 (10 μL) or SP (10 μL) samples were cast on the surface of the clean GCE respectively and thoroughly dried at room temperature. Then the GCE was placed into the electrolytic tank to test cyclic voltammetry curves.

Fluorescent and Photothermal Assay Procedure of β-Gal Activity

The procedure for β-Gal activity assay was as follows: 100 μL PAPG (15 mM), 90 μL 10 mM PIPES buffer (pH 7.3), and 10 μL of a fixed concentration of β-Gal solution were mixed in a 0.5 mL centrifuge tube. Then, the solution was incubated in a constant temperature oscillator (1,000 rpm) at 37°C for 80 min. Afterward, 15 μL diethylenetriamine (DETA) (20%, *v*/*v*) ethanol solution was added into the above tube. For the photothermal assay, the resulting solution was incubated at room temperature for 30 min, then irradiated for 3 min by taking advantage of 808 nm laser (1.5 W cm^{-2}). After irradiation, the temperature of the solution was recorded by using a smartphone-assisted portable infrared imager. For fluorescence assay, the resulting solution was incubated in a constant temperature oscillator (1,000 rpm) at room temperature for 50 min, then 4 μL CdTe@SiO_2 was added into the mixture. Finally, the mixture was

transferred to a cuvette to scan the fluorescence spectrum (λ_{ex} = 380 nm). The intensity ratio of 505 and 657 nm was recorded.

Fluorescent and Photothermal Assay Procedure of β-Gal Activity in Serum Samples

Human serum samples of twenty ovarian cancer patients and six healthy adults were collected from the hospital and stored at -20°C before use. To prepare a blank sample, the serum was diluted 5-fold with PIPES buffer (10 mM, pH 7.3). To inactivate the original β-Gal in serum, concentrated hydrochloric acid was added to the diluted serum with magnetic stirring for 30 min (pH 2). Then, the diluted serum pH was adjusted to 7.3 with NaOH aqueous solution (10 M). The obtained blank serum was used to prepare β-Gal-spiked serum samples, which were used to establish the working curve for the quantification of β-Gal in human serum samples. The procedure in the above section was performed except for replacing different concentrations of β-Gal buffer solution with 50 μL of β-Gal-spiked serum or 10 μL of real serum sample, respectively, and the added amount of PIPES buffer (10 mM, pH 7.3) was adjusted to ensure the same volume of reaction solution. The study was approved by the Medical Ethical Committee of Zhongnan Hospital of Wuhan University (approval 2023050K).

Cell Culture and Animal Model

MRC-5 cells, Vero cells, HeLa cells, MCF-7 cells, A549 cells, and SKOV-3 cells were all provided by the China Center for Type Culture Collection. Among these, MRC-5

cells, Vero cells, HeLa cells, MCF-7 cells and A549 cells were grown in Dulbecco's modified Eagle's media (DMEM) medium supplemented with 10% heat-inactivated FBS and 1% double antibiotic (penicillin-streptomycin). SKOV-3 cells were grown in McCoy's 5A medium containing 10% heat-inactivated FBS and 1% double antibiotic (penicillin-streptomycin). All cells were cultured in a 5% CO_2 humid incubator at 37°C.

Female BALB/c nude mice (4–5 weeks old) were purchased from SJA Laboratory Animal Co., Ltd (Hunan, China). SKOV-3 cells suspension (~1×10^7 in 200 μL for each nude mouse) was injected into the inguen of female nude mice to establish hypodermic SKOV-3 tumor models. Tumor-bearing nude mice were used for subsequent experiments until the tumor volume increased close to 110 mm^3. The tumor volume was calculated using the formula: Tumor volume = (length) × $(width)^2$ × 0.5.

Cell Viability Evaluation Assay

Cell viability was determined by cell counting kit-8 (CCK-8) assay. Vero and MRC-5 cells were seeded at a density of 5×10^3 cells per well in 96-well microplates with 200 μL cell suspension per well and cultured overnight in a 5% CO_2, 37°C humid incubator, resulting in a cell density of 80–90%. The original medium was aspirated and processed in three groups, as follows: (a) 250 μL fresh medium per well with the substrate PAPG concentration at 0.5, 1, 2, 3, 4 mg mL^{-1}; (b) 250 μL fresh medium per well with the DETA concentration at 0.16‰, 0.24‰, 0.32‰, 0.4‰, 0.48‰ (*v*/*v*); (c) The cells were incubated in fresh medium containing substrates of different concentrations (0.5, 1, 2,

3 and 4 mg mL^{-1}) for 2 h, and then DETA was added to make the concentrations of 0.16‰, 0.24‰, 0.32‰, 0.4‰ and 0.48‰ (*v*/*v*). For the control group, 250 μL fresh medium was added to each well, and no other reagents were added. After 12 hours of incubation, the culture medium was replaced with 100 μL of fresh medium containing 10% CCK-8 reagent, then co-culture for an additional 2 h. The absorbance at 450 nm was measured by the microplate reader. Cell viability (%) was calculated as: (OD_{450} test/ OD_{450} control) × 100%.

Fluorescent staining was used to determine the survival state of Vero and MRC-5 cells. The cells were cultured at 5% CO_2, 37°C in confocal dishes. Primary medium was removed after cells were adherent and then cells were washed twice with PBS buffer. The cells were incubated with 500 μL fresh medium containing substrate (2 mg mL^{-1}) for 2 h, then DETA was added to make the concentrations of 0.32‰, and further culture for 6 h. The cells were washed twice with PBS buffer, then incubated in 500 μL staining working solution (2 μM Calcein AM and 4.5 μM propidium iodide (PI)) for 20 min at room temperature in the dark. Cells images were acquired using Leica-LCS-SP8-STED with a 63×objective. Green channel at 500–550 nm with λ_{ex} = 488 nm, red channel at 600–760 nm with λ_{ex} = 561 nm.

Fluorescence Imaging in Living Cells

SKOV-3 cells were cultured overnight in confocal dishes at 37°C, 5% CO_2 and primary medium was removed after cells were adherent. For the experimental group, the cells were cultured with 500 μL of 2 mg mL^{-1} substrate (10 mM PIPES buffer: phenol red-

free DMEM (2:1, *v*/*v*) as solvent) for 2 h, then DETA was added to make the concentrations of 0.32‰ and incubated for 1 h before imaging. For the inhibitor-treated group, the cells were incubated with 1 mM D-galactose for 1 h, and the subsequent operations were the same as those of the experimental group. As control cells, MRC-5 cells, Vero cells, HeLa cells, MCF-7 cells, and A549 cells were treated in the same manner as the SKOV-3 experimental group. Before confocal imaging, the primary medium was removed, and the cells were washed twice with PBS. All cells were imaged with a laser scanning confocal microscope at 405 nm, and the emission signal in the range of 460–580 nm was collected.

Synthesis of OM for Photothermal Therapy

10 μL of 20% (*v*/*v*) DETA solution and 40 μL *p*-AP (10 mM) were added in 960 μL PBS buffer (DETA and *p*-AP were prepared with PBS buffer solution with pH 7.4), the resulting solution was incubated for 1 h at room temperature.

Cell Viability Evaluation of Synthetic OM

The cell biocompatibility of OM was studied by CCK-8 assay. Vero cells inoculated on 96-well plates were cultured in cell incubators for 12 h. The cell culture liquid was changed with fresh DMEM and different volumes of OM. After 12 h incubation, the culture medium was replaced with 100 μL of fresh medium containing 10% CCK-8 reagent, then co-cultured for an additional 2 h. The absorbance was measured at 450 nm using the microplate reader. Cell viability (%) was calculated as: (OD_{450} test/OD_{450}

control) × 100%.

In Vitro Cytotoxicity of Synthetic OM

CCK-8 assay was used to evaluate the cytotoxicity of OM on ovarian cancer cells. SKOV-3 cells were seeded at a density of 5×10^3 cells per well in 96-well microplates with 200 μL cell suspension per well and cultured overnight in cell incubators. Then the primary medium was replaced with 200 μL DMEM, adding different volumes of OM, and the cells were incubated for another 20 min. For the laser irradiation group, the cells were exposed to 808 nm laser (1.0 W cm^{-2}) for 5 min. After that, the cells were incubated for additional 12 h and their viability was analyzed using CCK-8 assay.

Fluorescent staining was used to determine the survival state of cells. SKOV-3 cells were cultured in a cell incubator, and treated with 500 μL DMEM adding 25 μL OM for 20 min in the presence or absence of 808 nm laser photoirradiation for 5 min with 1.0 W cm^{-2} laser power density. After further culture for 12 h, the cells were washed twice with PBS buffer, then incubated in 500 μL staining working solution (2 μM Calcein AM and 4.5 μM PI) for 20 min at room temperature in the dark. Cells fluorescence images were acquired using Leica-LCS-SP8-STED with a 20 × objective. Green channel at 500–550 nm with λ_{ex} = 488 nm, red channel at 600–760 nm with λ_{ex} = 561 nm.

Flow Cytometry

SKOV-3 cells were incubated in six-well plates in a cell incubator for 12 h. The cells

were incubated with 1 mL new DMEM and 100 μL OM for 20 min in the presence or absence of 808 nm laser irradiation for 5 min with 1.0 W cm^{-2} laser power density. After further culture for 24 h, the cells were digested with 200 μL of EDTA-free trypsin, and after centrifugation at 1,800 rpm and 4°C for 5 min, 100 μL of binding buffer was added to make single cell suspension. Subsequently, the above cells were further stained with an Annexin V-FITC/PI apoptosis kit to evaluate the cell apoptosis with a Cytoflex flow cytometer. The above cell experiments were conducted three replicates per group to guarantee accuracy.

In Vivo Photothermal Therapeutic Efficacy of Synthetic OM

SKOV-3 tumor-bearing nude mice were randomly divided into four groups (n = 3 per group): (a) PBS + Laser group, (b) OM injection group, (c) OM + Laser group, (d) ICG + Laser group. SKOV-3 tumor-bearing mice were anesthetized. Then, 50 μL PBS, OM, or ICG was injected into the tumor. For groups (a), (c), and (d), tumors were continuously irradiated under an 808 nm laser (1.0 W cm^{-2}) for 5 min. Finally, the real-time temperature was recorded every 30 s with an infrared camera. Each group was treated on day 1 and day 3. The size of the tumor was recorded every 2 days with a digital caliper during the whole treatment period (15 d), and the weight of the mice was recorded every 2 days. All animal procedures were carried out under the guidelines set by the Institutional Animal Care and Use Committee of Hubei Province, and the overall project protocols were approved by the Animal Ethics Committee of Wuhan University (No.WP20220227).

Immunohistochemical Staining Analysis

After 15 days of photothermal therapy, the major organs (heart, liver, spleen, lung, and kidney) were isolated from the tumor-bearing nude mice and analyzed by H&E assay for biocompatibility evaluation, and serum samples were collected for biochemical tests.

Acute Toxicity Experiment

After fasting for 12 hours, referring to the range of single dose volume in mice (0.05–0.10 mL/10 g), nude mice were intravenously injected with different volumes of OM solution (50 μL, 100 μL, 150 μL, and 200 μL), three nude mice per group. The control group received 200 μL PBS. After treatments, the animals were observed in the first 12 h, toxic signs of a general nature were observed and registered. After this period, the animals were observed for up to 7 days and were weighed daily after treatments. At the end of the observation period, all the survivors were euthanized and autopsied. The macroscopic characteristics of the major organs (heart, liver, spleen, lung, and kidney) were observed. Additionally, serum samples were collected for biochemical tests. All animal procedures were carried out under the guidelines set by the Institutional Animal Care and Use Committee of Hubei Province, and the overall project protocols were approved by the Animal Ethics Committee of Wuhan University (No.WP20220227).

Evaluation of Genotoxicity by Single-Cell Gel Electrophoresis Assay (Comet Assay)

The evaluation of genotoxicity of OM was studied by comet assay kit. MRC-5 cells

were incubated with 2 mL DMEM in six-well plate for 12 h. The cells (1×10^6 cells mL^{-1}) were incubated with different doses of OM (40 μL and 60 μL). Methyl methanesulfonate (MMS) (40 μL, 0.25 μg mL^{-1}) and PBS (40 μL) were used as positive and negative controls, respectively. After further culture for 6 h, the cells were digested with 200 μL of EDTA-free trypsin, and after centrifugation at 1,800 rpm and 4°C for 5 min, 50 μL of PBS buffer was added to make single cell suspension. Then, the cells were mixed with 0.75% low melting point agarose in PBS buffer and added to slides pre-coated with a layer of 1% normal melting point agarose in PBS. Agarose was allowed to set at 4°C for 10 min and then the slides were immersed in ice-cold freshly prepared lysis solution overnight at 4°C. Electrophoresis was carried out in freshly prepared pre-chilled buffer (0.2 M NaOH, 1 mM EDTA) for 20 min at 25 V. The slides were neutralized with Tris-HCl buffer (pH 7.5) for 5 min and stained with 20 μL propidium iodide solution for 20 min. They were then washed with ultrapure water, covered with a cover slip, and visualized under a fluorescence microscope.

Statistical Analysis

Experiments were performed with at least three replicates, and all quantitative data are presented as the means ± SD. Statistical analysis was performed with Origin software. Comparison of multiple groups was performed using analysis of variance (ANOVA). Statistical significance is represented as $^{**}p < 0.01$; $^{***}p < 0.001$.

Partial Results and Discussion

Synthesis and Characterization of CdTe and CdTe@SiO_2

CdTe was synthesized by the solvothermal method (Figure S15), and the synthesis condition (the volume ratio of TEOS to CdTe) of CdTe@SiO_2 was investigated. The fluorescence signals of different CdTe@SiO_2 are almost the same, illustrating that the coating of silica does not affect the fluorescence of CdTe (Figure S16). As shown in Figure S17A, when the volume ratio of TEOS to CdTe is 1:1000, CdTe is not completely covered by silicon dioxide. As seen in Figure S17C-E, CdTe QDs shows obvious agglomeration phenomenon, resulting in uneven QDs coating due to high local concentration and fast hydrolysis of TEOS. Therefore, the optimal volume ratio of TEOS to CdTe is 2:1000 (Figure S17B). The TEM results show the morphology of the CdTe and CdTe@SiO_2 (Figure S18A,B), and CdTe exhibits uniform spherical structure, and the average particle size is approximately 3.21 nm. As seen in Figure S18B, SiO_2 coating completely covers the surface of the CdTe. The FT-IR spectroscopies further confirm the successful synthesis of CdTe (Figure S18C). The peaks at 3432.67 cm^{-1} of CdTe and 3434.60 cm^{-1} of CdTe@SiO_2 are attributed to stretching vibration of -OH, the peak values of 1384.64 cm^{-1} to 1627.63 cm^{-1} are the symmetry and asymmetry stretching vibration of COO^-. The peak at 2501.22 cm^{-1} is attributed to the stretch vibration peak of -SH in NAC. The disappearance of this peak in CdTe indicates that the binding of NAC and CdTe is through the coordination of S on sulfhydryl group and Cd on CdTe to form Cd-S bond. Additionally, the peak at 1106.94 cm^{-1} corresponds to the Si-O asymmetric stretching of CdTe@SiO_2. The FT-IR results confirm the successful synthesis of the CdTe and CdTe@SiO_2. The XPS results demonstrate that

SiO_2 is successfully coated on CdTe because the diffraction peak of Si is observed in XPS spectra (Figure S18D and Figure S19).

The optical properties of CdTe and CdTe@SiO_2 were characterized using the UV-vis absorption and fluorescence spectra. As shown in Figure S20, the absorption maximum of the solution of CdTe (622 nm) and CdTe@SiO_2 (623 nm) are almost the same. The excitation and emission spectra of CdTe@SiO_2 are shown in Figure S21, when excited by the maximum excitation wavelength of 380 nm, the maximum emission wavelength is 657 nm. The absolute quantum yield (QY) of CdTe and CdTe@SiO_2 is found to be 23.89% and 29.24%, respectively, indicating that the SiO_2 coating improves the absolute QY of CdTe. Additionally, the fluorescence intensity of CdTe@SiO_2 is continuously monitored for 1,800 s by fluorescence spectrometer, and it remains stable (Figure S22).

Exploration of Fluorescence Turn-off Mechanism

The fluorescence turn-off mechanism of CdTe@SiO_2 by SP was investigated. As shown in Figure S25, cyclic voltammetry is used to determine the oxidation potential (E_{ox}) of CdTe@SiO_2 with reference in the literature. The energy of the conduction bands for CdTe@SiO_2 was calculated to be -5.869 eV according to the Eqs. 3 and 4. As shown in Figure S20, CdTe@SiO_2 possesses a maximum absorption edge at 684.2 nm, and the band-gap energy (E_g) of 1.81 eV was worked out according to the formula of $E_g = 1240/\lambda_{abs}$, and the energy of the valence bands was estimated to be -4.059 eV on the base of Eq. 5. The E_{ox} and E_{red} of SP were 0.26 eV and -0.34 eV, respectively. The

conduction band and valence band were calculated to be -5.559 eV and -4.959 eV, and the conduction band is lower than that of CdTe@SiO_2 (-5.869 eV), indicating that energy can transfer from CdTe@SiO_2 to SP. Therefore, the fluorescence turn-off response of CdTe@SiO_2 is achieved by energy transfer. Additionally, As seen in Figure 3C, the Zeta potential of SP and CdTe@SiO_2 is found to be -22.0 mV and -11.9 mV in PIPES buffer (10 mM, pH 7.3), respectively, while the Zeta potential of the mixture of SP and CdTe@SiO_2 is -11.7 mV, indicating that SP and CdTe@SiO_2 tend to interact with each other. Then, the fluorescence lifetime of CdTe@SiO_2 and the mixture of SP and CdTe@SiO_2 was determined. The decay curves are shown in Figure 3C, the fluorescence lifetime is 90.39 ns and 74.90 ns of CdTe@SiO_2 and the mixture of SP and CdTe@SiO_2, respectively. The decrease of the fluorescence lifetime of the mixture indicates that the mechanism of fluorescence quenching is dynamic quenching, which results from the molecular diffusion and collision.

$$E_{CB} = -e(E^{ox}_{onset\rightarrow ref} + 4.5V) = -e\ (E_{ox} + E_{ref} + 4.5V) \quad (3)$$

$$E_{Ag/Ag+}{}^{ref} = 0.799\ V \quad (4)$$

$$E_{VB} = E_{CB}\text{-}E_g \quad (5)$$

Optimization of Reaction Parameters of Dual-Mode Assay

Several parameters that influence the detection performance were optimized, including pH of the PIPES buffer in enzymatic reaction, enzymatic reaction time, concentration of PAPG, volume of DETA (20%, *v*/*v*) ethanol solution, polymerization reaction time, volume of CdTe@SiO_2, and laser irradiation time. The variation of temperature (ΔT),

the variation of F_{505nm}/F_{657nm}, and the chromaticity shift value between the experimental group (with 80 U L^{-1} β-Gal) and blank group (without β-Gal) were chosen as the criteria to determine the optimal conditions. First, the pH of PIPES buffer was also investigated. According to Figure S26A, ΔT and $\Delta(F_{505nm}/F_{657nm})$ reach the maximum value at 7.3 and begin to decrease at a higher pH value, thus the optimal pH of PIPES buffer (10 mM) in enzymatic reaction is set at 7.3. The enzymatic reaction time was examined next (Figure S26B). When the reaction time is extended to 80 min, both ΔT and $\Delta(F_{505nm}/F_{657nm})$ reach the highest level. Thus, the optimum enzymatic reaction time of 80 min is selected for the following assays. In addition, the concentration of PAPG was optimized. As shown in Figure S26C, the maximum ΔT and $\Delta(F_{505nm}/F_{657nm})$ are obtained at 7.5 mM. Seen in Figure S26D, the optimal volume of DETA (20%, *v*/*v*) ethanol solution is 15 μL. Furthermore, the polymerization reaction time of fluorescent method and photothermal method are 50 min and 30 min, respectively (Figure S26E). For photothermal analysis, the results (Figure S26F) indicate that the optimal time is 3 min. For fluorescent analysis, the volume of CdTe@SiO_2 was investigated. As seen in Figure S27, $\Delta(F_{505nm}/F_{657nm})$ shows a decreasing trend between 1–5 μL. In this case, the chromaticity shift value can help to correct the results and achieve dual emission fluorescence. It reaches the maximum at 4 μL, which is the optimal volume of CdTe@SiO_2.

Optimization of OM Synthesis Parameters

The synthesis conditions of OM (including concentration of *p*-AP, volume of DETA,

and polymerization reaction time) were optimized to obtain better photothermal effects. The temperature of OM was used as an indicator to determine the optimal synthesis conditions. Excessive concentration of DETA can cause damage to cells, so we fixed the concentration of DETA at 2% (*v*/*v*). First, we investigated the concentration of *p*-AP, as shown in Figure S34A,B, and the maximum temperature is obtained at 400 μM. Therefore, the concentration of *p*-AP is set as 400 μM. As seen in Figure S34C,D, the temperature increases rapidly when the polymerization reaction time is from 0.5 to 1 h, and then it does not change much as the reaction time continues to extend. Thus, the reaction time is set as 1 h.

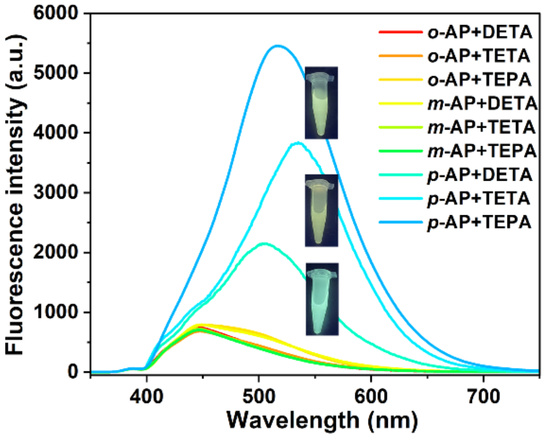

Figure S1. Screening results of monomers. The fluorescent spectra of different solutions. The preparation of SP: 50 μL crosslinking agents (DETA, TETA, and TEPA) (20%, *v*/*v*) ethanol solution was mixed with 1 mL monomer (*o*-AP, *m*-AP, or *p*-AP, 200 μM) in PIPES buffer (10 mM, pH 7.3) and reacted at room temperature for 30 min.

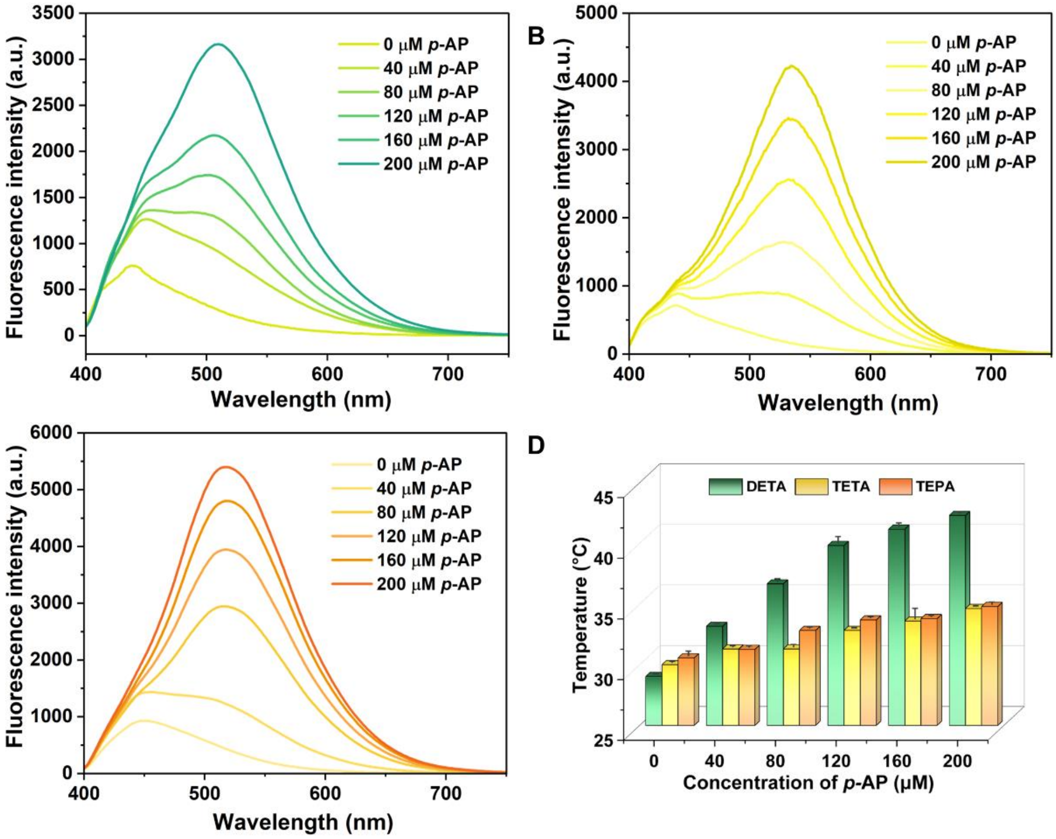

Figure S2. Screening results of crosslinking agents. The variation of reaction solution fluorescence versus the concentration of *p*-AP (0–200 μM), the crosslinking agent is DETA A), TETA B), and TEPA C), respectively. D) The variation of reaction solution temperature versus the concentration of *p*-AP (0–200 μM). The preparation of reaction solution: 50 μL crosslinking agents (DETA, TETA, and TEPA) (20%, *v*/*v*) ethanol solution was mixed with 1mL different concentrations of *p*-AP in PIPES buffer (10 mM, pH 7.3) and reacted at room temperature for 30 min, respectively.

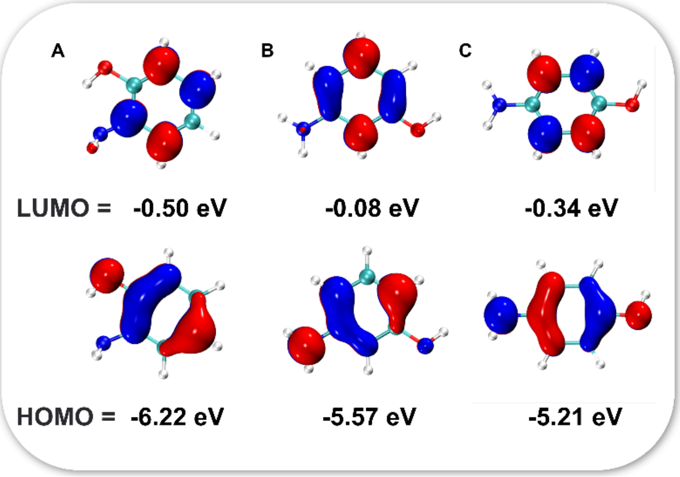

Figure S3. Theoretical calculation results of monomer molecules. The frontier molecular orbital (the highest occupied molecular orbital and lowest unoccupied molecular orbital, HOMO and LUMO) diagrams of *o*-AP A), *m*-AP B) and *p*-AP C) obtained by Multiwfn and VMD.

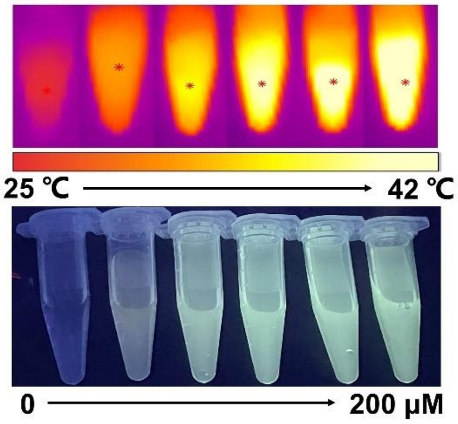

Figure S4. The infrared thermal and fluorescence image of reaction solution. The preparation of reaction solution: 50 μL DETA (20%, *v*/*v*) ethanol solution was mixed with 1 mL different concentrations (0–200 μM) of *p*-AP in PIPES buffer (10 mM, pH 7.3) and reacted at room temperature for 30 min, respectively. The fluorescence image was obtained under the excitation of 365 nm UV light, and the infrared thermal image was obtained under the irradiation of 808 nm laser (1.5 W cm^{-2}) for 3 min.

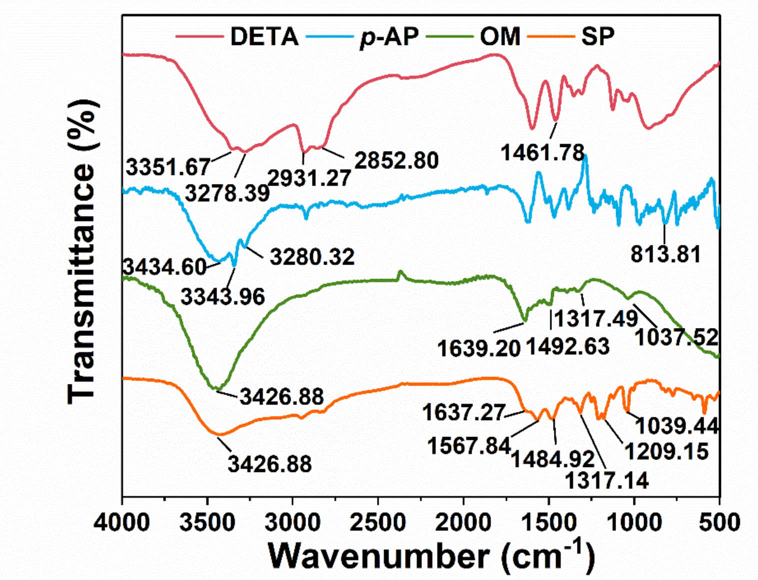

Figure S5. FT-IR spectra of DETA, *p*-AP, OM, and SP.

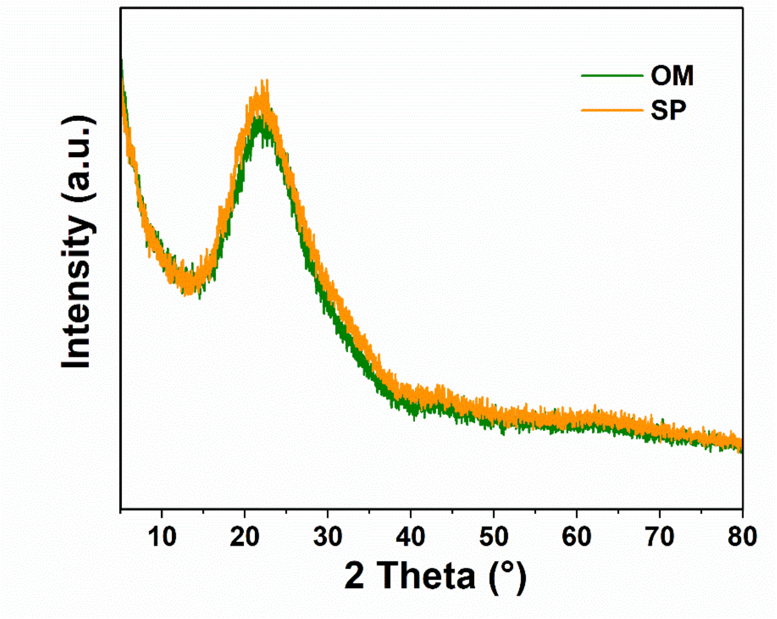

Figure S6. PXRD pattern of OM and SP.

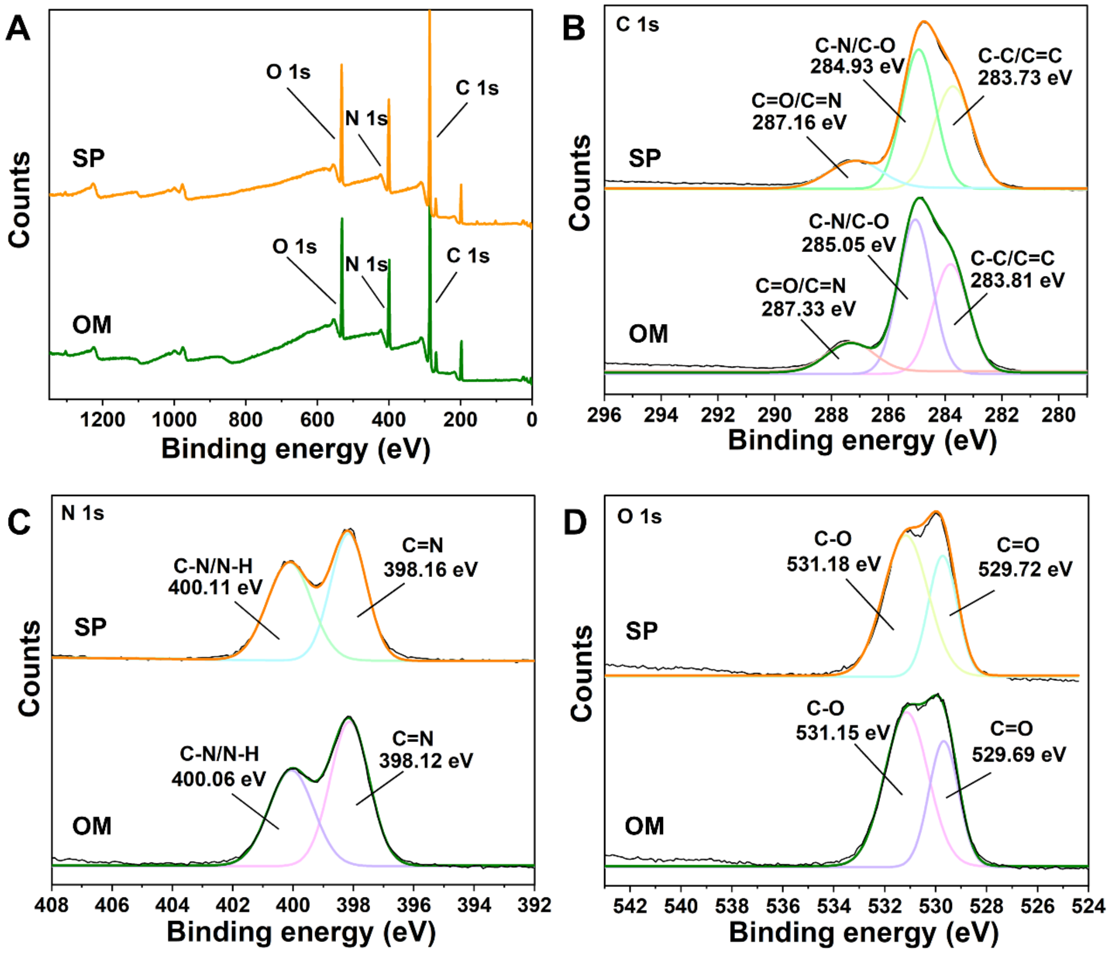

Figure S7. XPS results of OM and SP. A) The survey scan of OM and SP. The refinement spectra of C 1s B), N 1s C), and O 1s D).

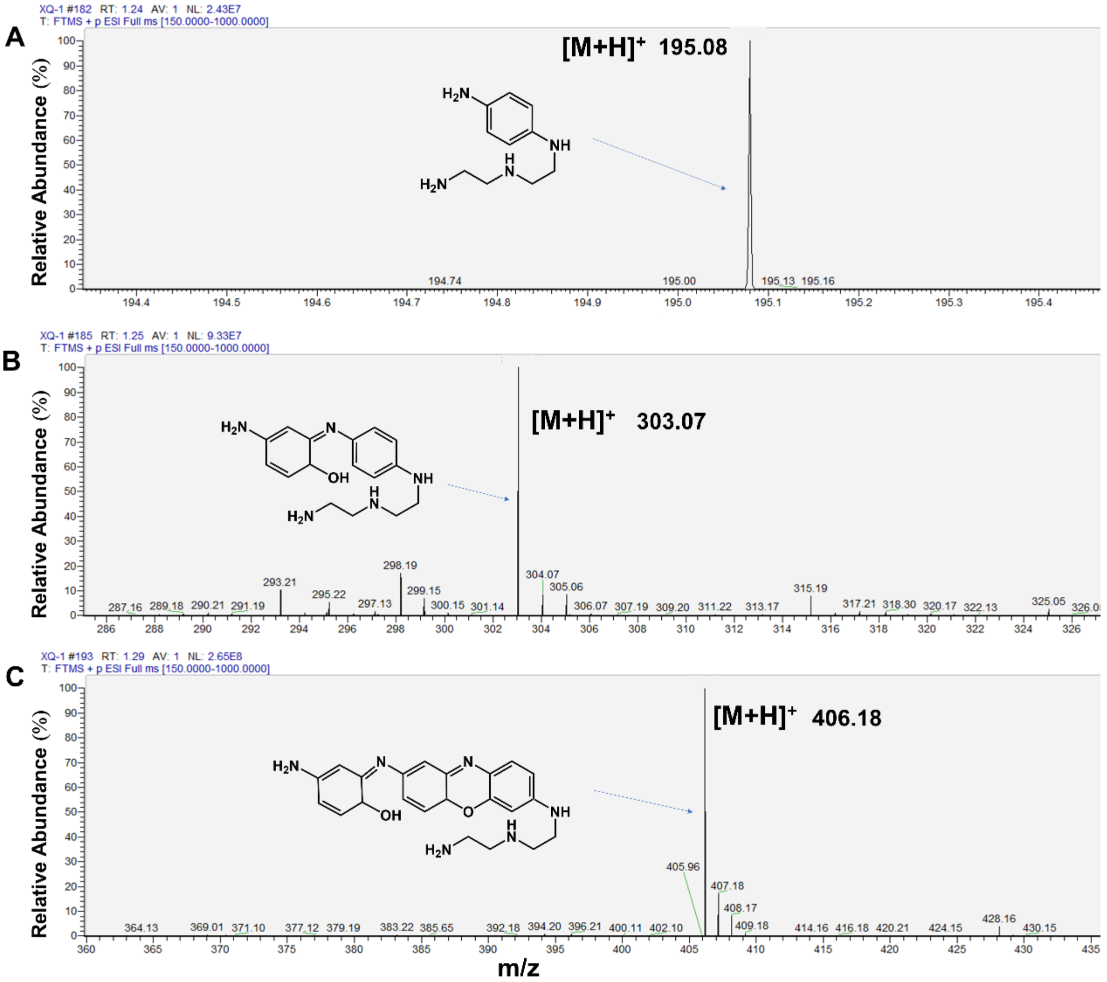

Figure S8. The mass spectra of OM solution. 75 μL of DETA (20%, *v*/*v*) ethanol solution was mixed with 1 mL *p*-AP (200 μM) in PIPES buffer (10 mM, pH 7.3), reacting for 30 min at room temperature. The solution was diluted 500-fold and analyzed by ultra-high resolution mass spectrometer (Q Exactive, Thermo Scientific) with positive electrospray ionization mass spectrometry (ESI-MS) analysis.

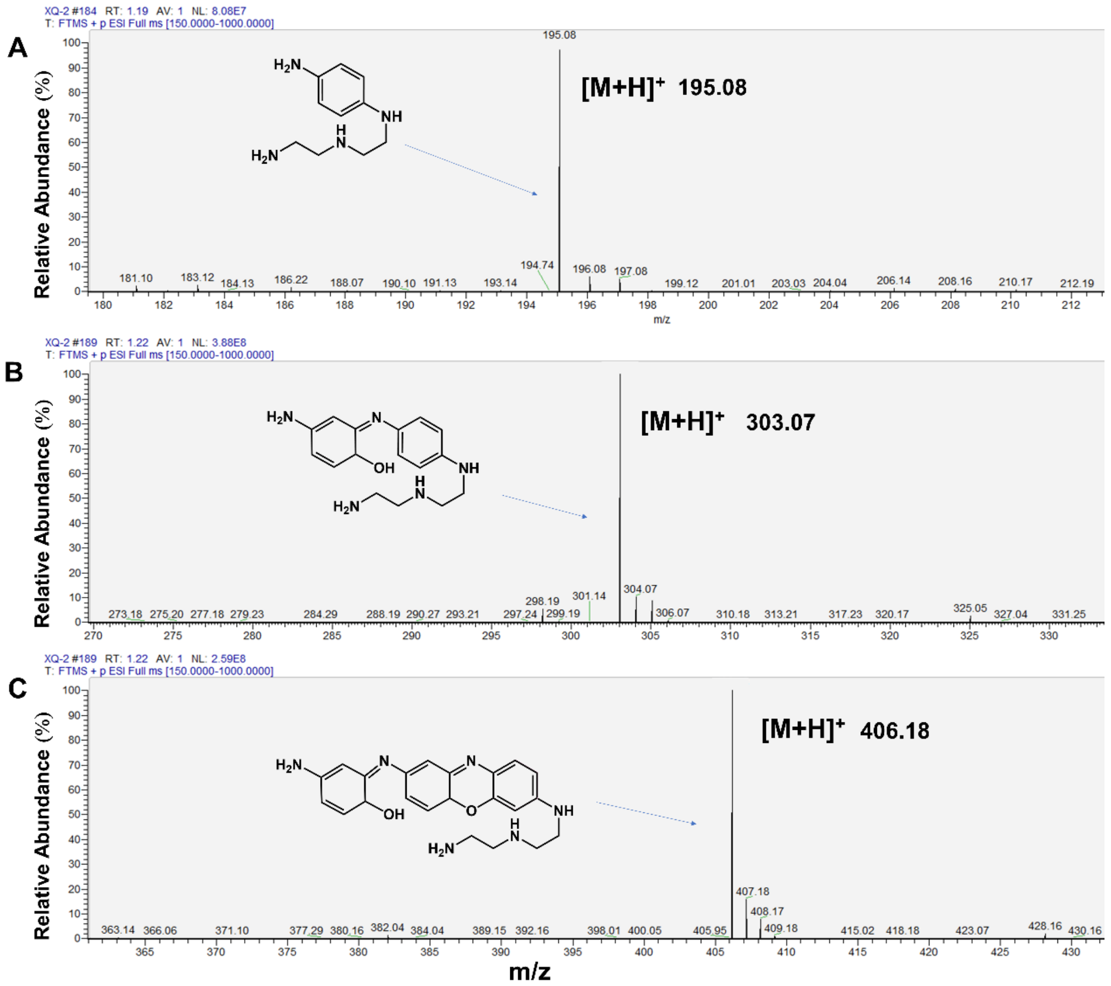

Figure S9. The mass spectra of SP solution. 75 μL of DETA (20%, *v*/*v*) ethanol solution was mixed with 1 mL *p*-AP (200 μM) in PIPES buffer (10 mM, pH 7.3), reacting for 50 min at room temperature. The solution was diluted 500-fold and analyzed by ultra-high resolution mass spectrometer (Q Exactive, Thermo Scientific) with positive electrospray ionization mass spectrometry (ESI-MS) analysis.

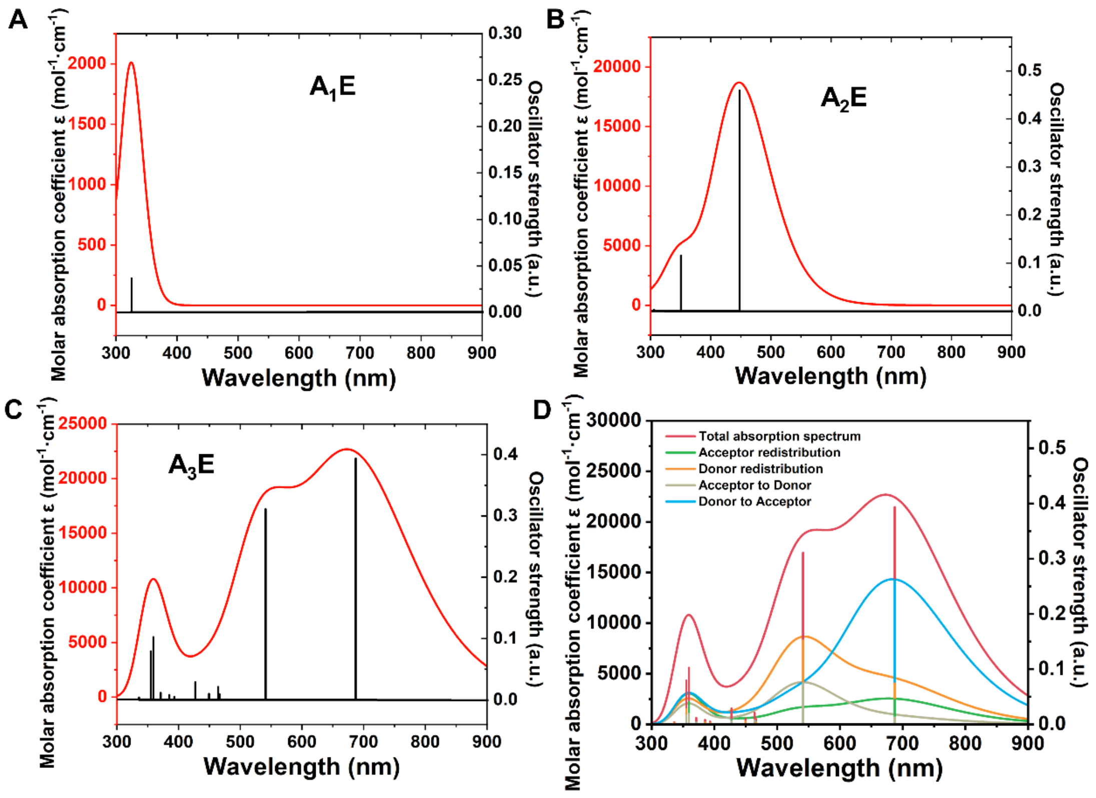

Figure S10. The electronic absorption spectra of the molecules. The absorption spectra of A_1E A), A_2E B), and A_3E C) were calculated by Multiwfn. D) Electronic absorption (red) and charge-transfer spectra (CTS) of A_3E.

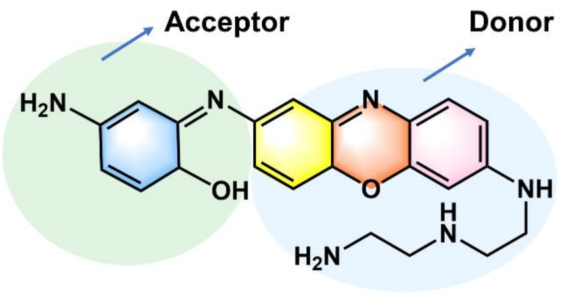

Figure S11. Distribution diagram of donor and acceptor in A_3E.

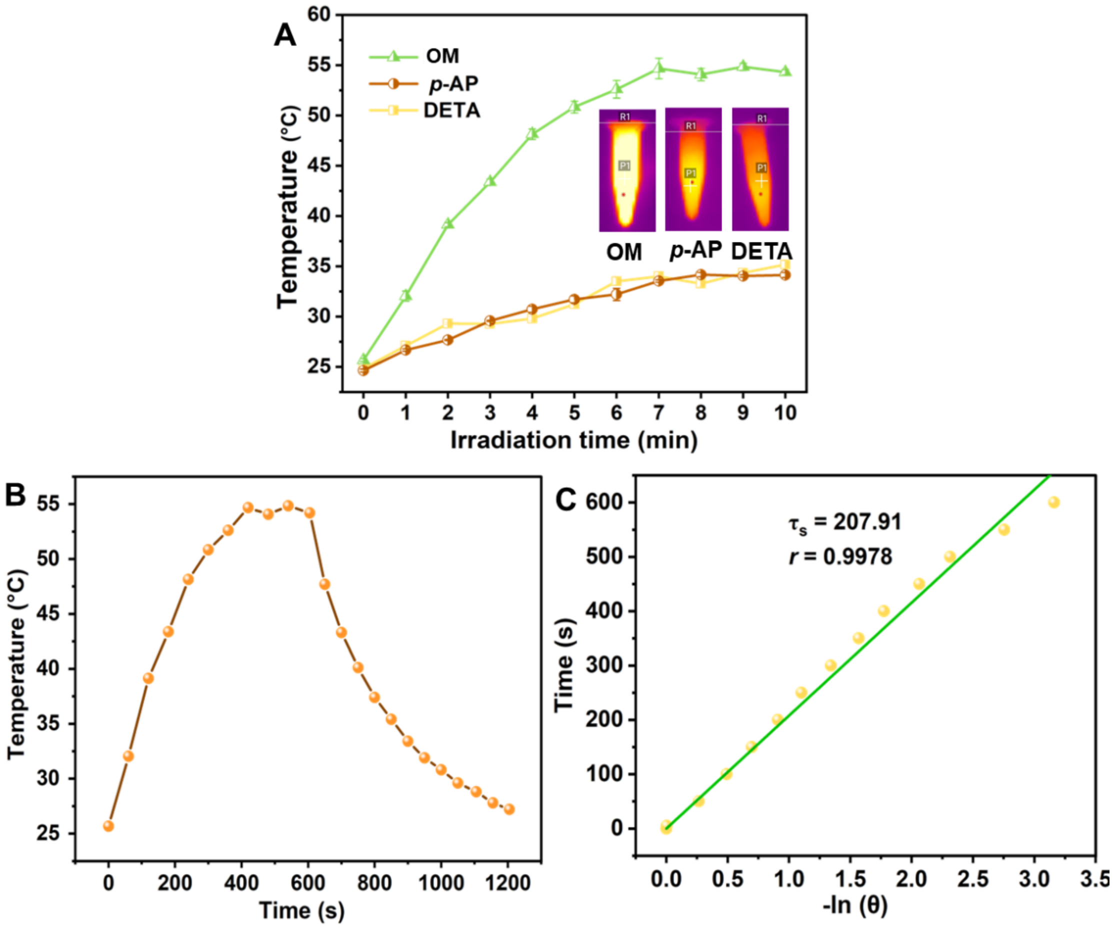

Figure S12. Photothermal property of OM. A) The temperature curves of OM, *p*-AP and DETA under 808 nm laser irradiation (1.5 W cm^{-2}) for 10 min and the corresponding infrared thermal images at the highest temperature. The preparation of OM: 75 μL DETA (20%, *v/v*) ethanol solution was mixed with 1 mL *p*-AP (200 μM) in PIPES buffer (10 mM, pH 7.3), reacting for 30 min at room temperature. B) Temperature profile of reaction system irradiated with 808 nm laser (1.5 W cm^{-2}), followed by natural cooling with the turn-off of the laser. C) Determination of system time constant (τ_s) through the linear regression of the cooling profile shown in B), according to Eq. 2, the slope of the linear equation is the τ_s values.

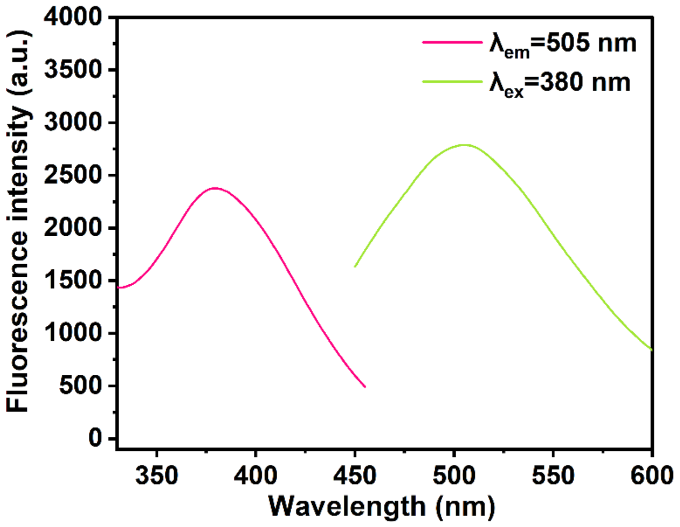

Figure S13. The fluorescence excitation (pink) and emission (green) spectra of SP.

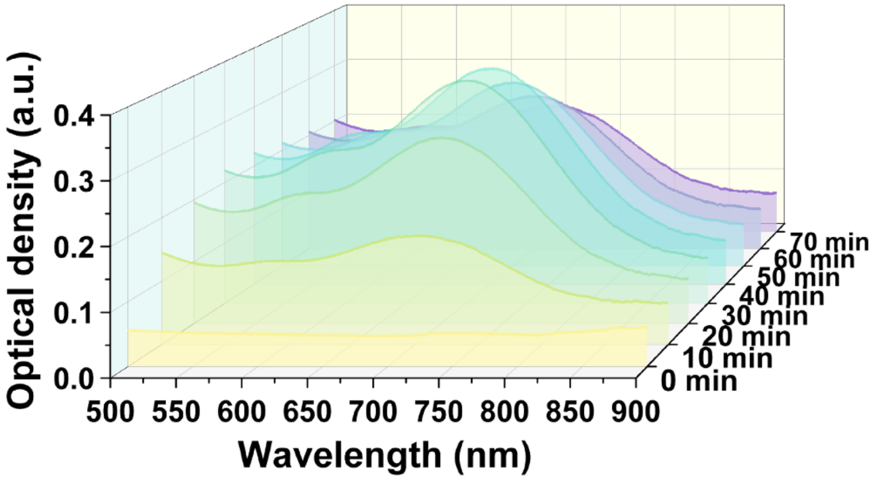

Figure S14. The absorption spectra of cascade polymers with the increase of polymerization time (0–70 min). Polymeric conditions: 75 μL of DETA (20%, *v/v*) ethanol solution was mixed with 1 mL *p*-AP (200 μM) in PIPES buffer (10 mM, pH 7.3), and polymerization time 0–70 min.

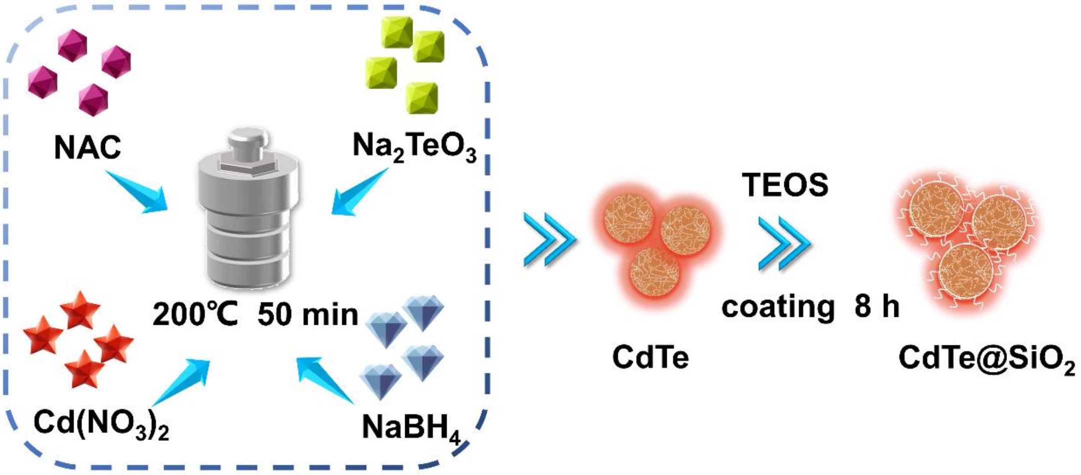

Figure S15. The schematic diagram for the synthesis of CdTe@SiO_2.

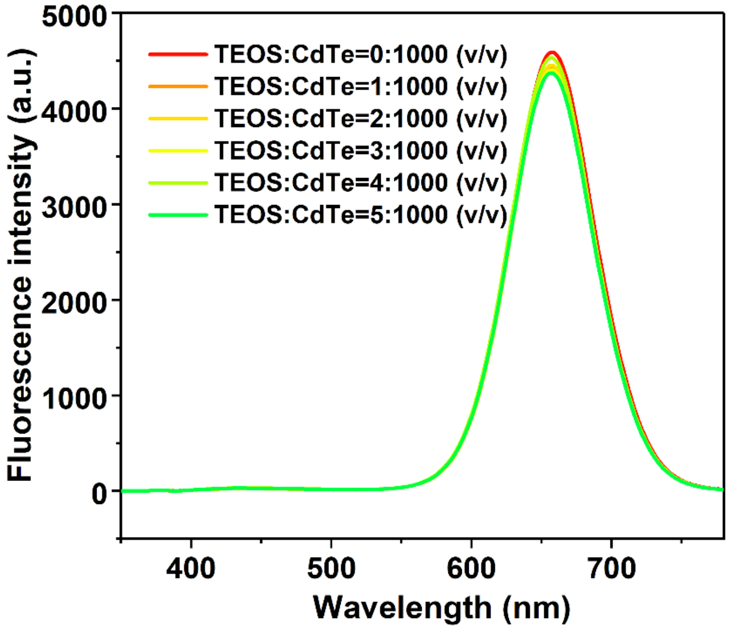

Figure S16. The fluorescence emission spectra of CdTe@SiO_2 with different synthesis conditions (λ_{ex}=380 nm).

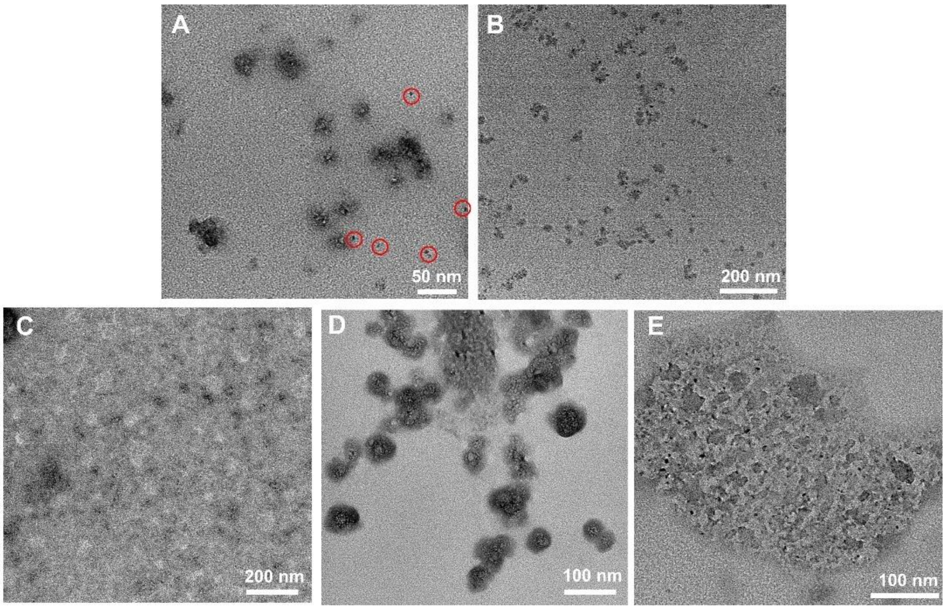

Figure S17. The TEM results of CdTe@SiO_2 with different synthesis conditions. TEOS: CdTe = 1:1000 (*v/v*) A), TEOS: CdTe = 2:1000 (*v/v*) B), TEOS: CdTe = 3:1000 (*v/v*) C), TEOS: CdTe = 4:1000 (*v/v*) D), and TEOS: CdTe = 5:1000 (*v/v*) E).

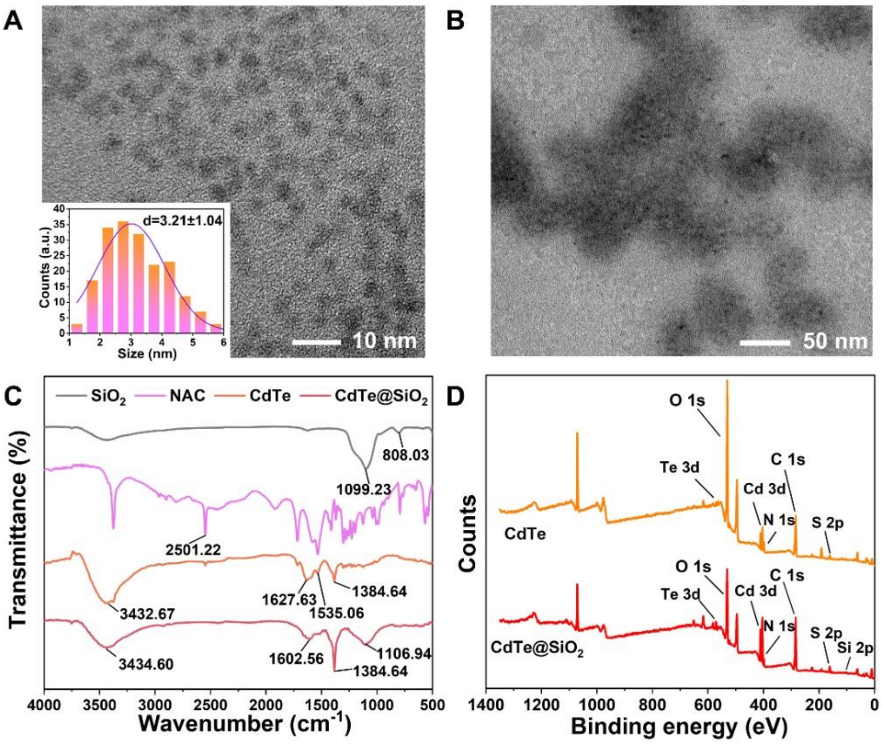

Figure S18. Characterization of CdTe and CdTe@SiO_2. TEM results of CdTe A) and CdTe@SiO_2 B). C) FT-IR spectra of SiO_2, NAC, CdTe and CdTe@SiO_2. D) XPS results of CdTe and CdTe@SiO_2.

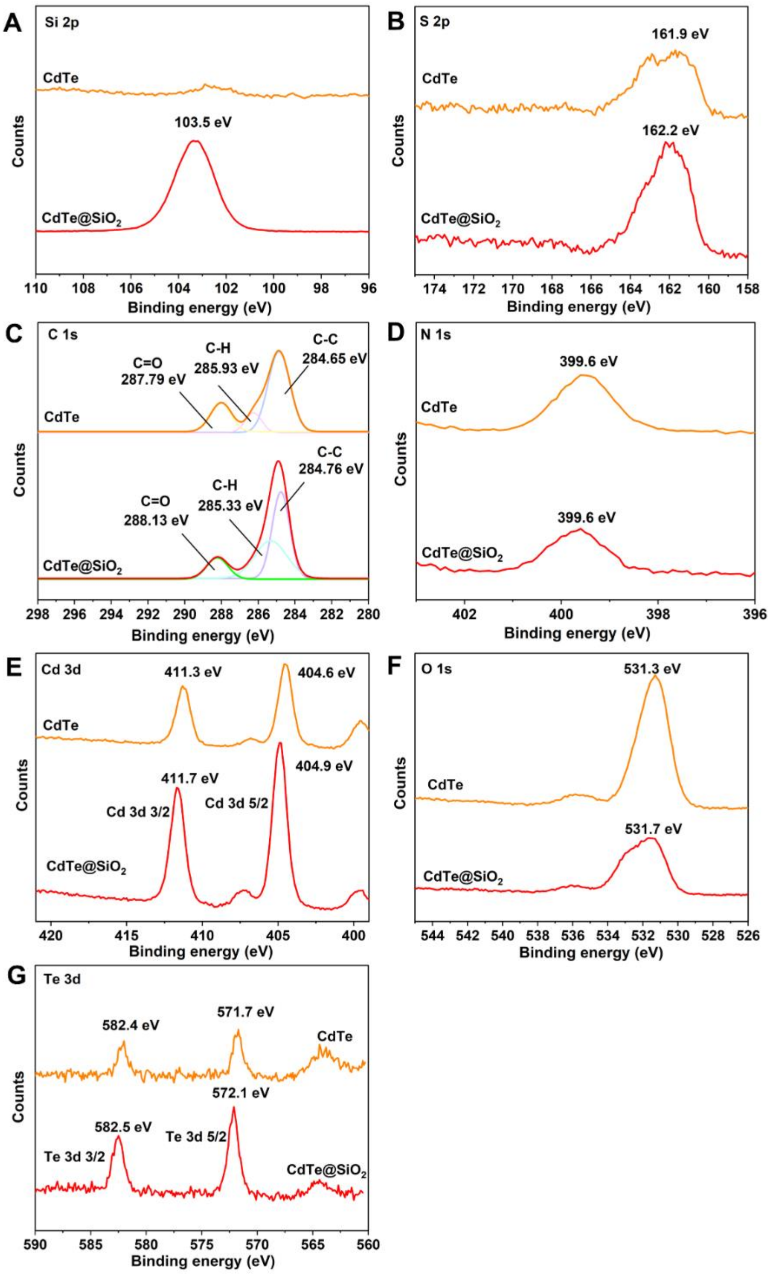

Figure S19. The XPS analysis of CdTe and CdTe@SiO_2. The spectra of Si 2p A), S 2p B), C 1s C), N 1s D), Cd 3d E), O 1s F), and Te 3d G).

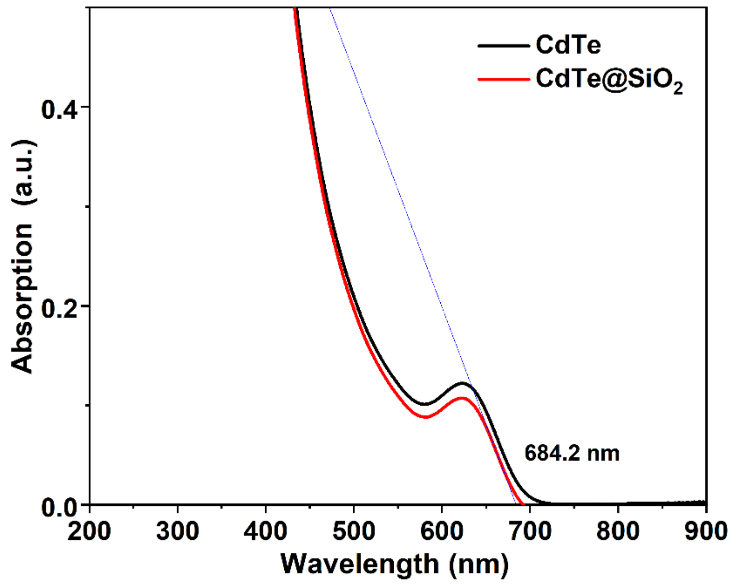

Figure S20. The absorption spectra of CdTe (black) and CdTe@SiO_2 (red).

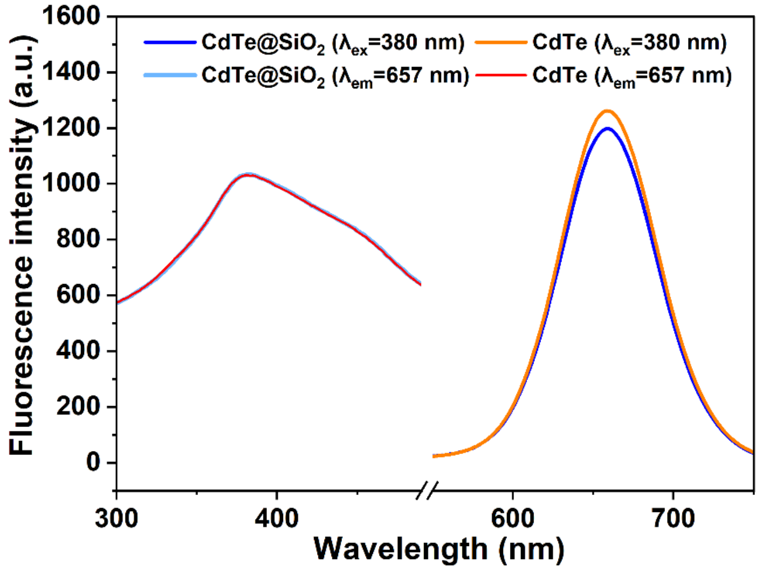

Figure S21. The fluorescence excitation and emission spectra of CdTe and CdTe@SiO_2.

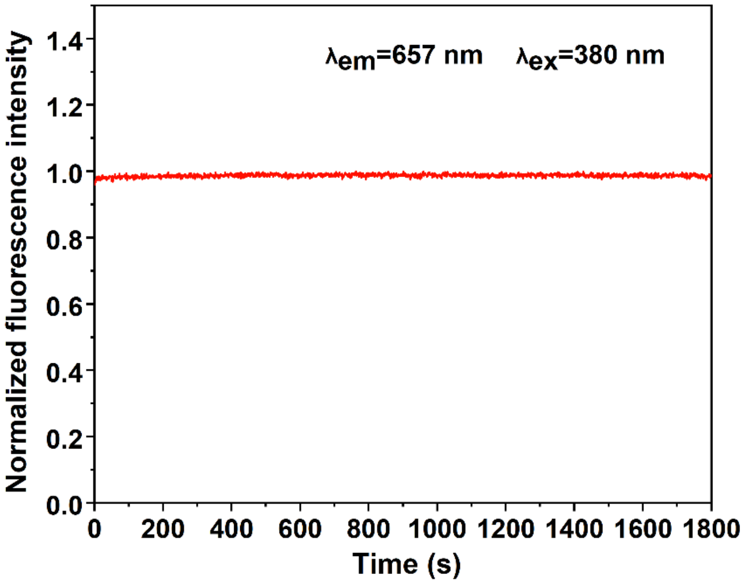

Figure S22. The fluorescence intensity stability of CdTe@SiO_2 within 1,800 s. Scan speed is 1,200 nm min^{-1}, EX slit is 5 nm, EM slit is 5 nm, PMT voltage is 700 V, and response time is 2 s.

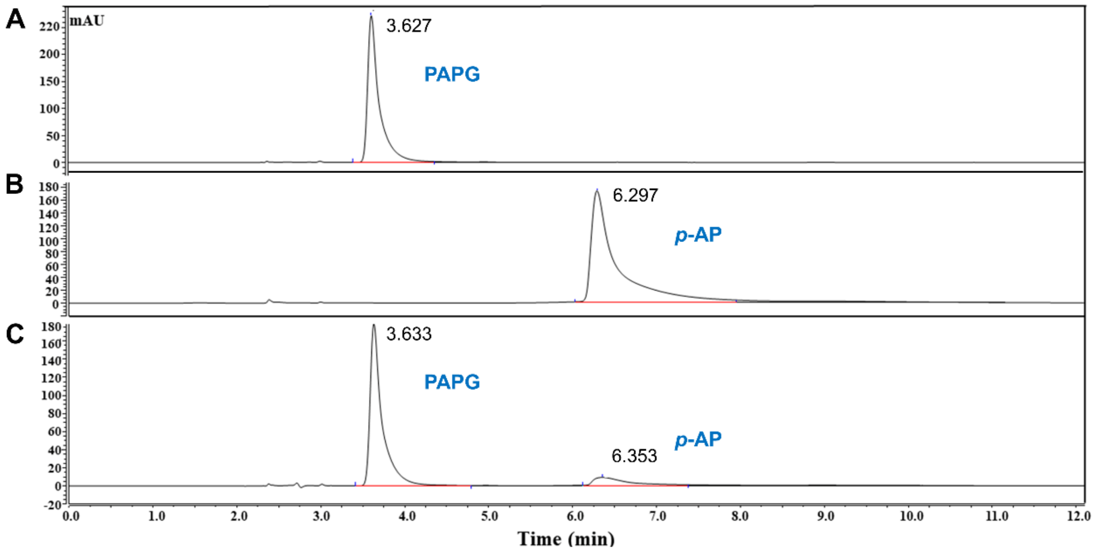

Figure S23. Verification results of β-Gal enzymolysis of substrate PAPG. HPLC chromatogram of A) PAPG (10 mM), B) *p*-AP (2 mM), and C) reaction solution (10 mM PAPG, 50 U L^{-1} β-Gal in 1 mL PIPES buffer (10 mM, pH 7.3)), which was incubated in a constant temperature oscillator (800 rpm) at 37°C for 60 min. Chromatographic separation of analytes was performed on Shimadzu Inertsil ODS (250 mm × 4.6 mm, 5 μm) at room temperature. The mobile phase was composed of 90% water and 10% methanol with an isocratic elution.

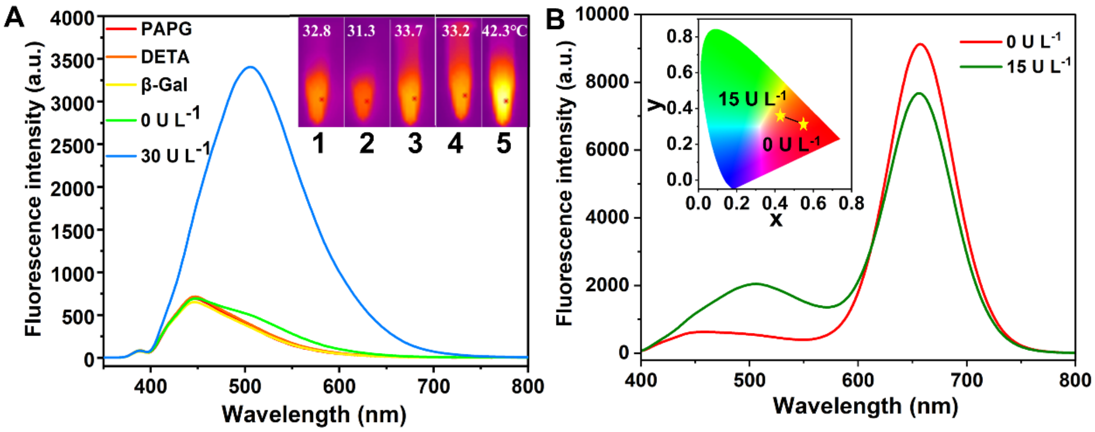

Figure S24. Results of β-Gal activity detection pathway verification. A) The fluorescent spectra of different solutions. Inset: infrared images of solutions, 1: PAPG solution (5 mM PAPG in 10 mM PIPES buffer, pH 7.3), 2: DETA solution (20 μL DETA (20%, *v*/*v*) ethanol solution in 10 mM PIPES buffer), 3: β-Gal (100 U L^{-1} in 10 mM PIPES buffer), 4 and 5: enzyme reaction system without β-Gal/with β-Gal (30 U L^{-1}). B) The fluorescence spectra and the CIE chromaticity diagram of the reaction solutions with CdTe@SiO_2. 0 U L^{-1} β-Gal (Blank group) and 15 U L^{-1} β-Gal (Experimental group). Other conditions: enzymatic reaction time is 60 min, polymerization time of photothermal and fluorescent assay are 30 min and 60 min, respectively.

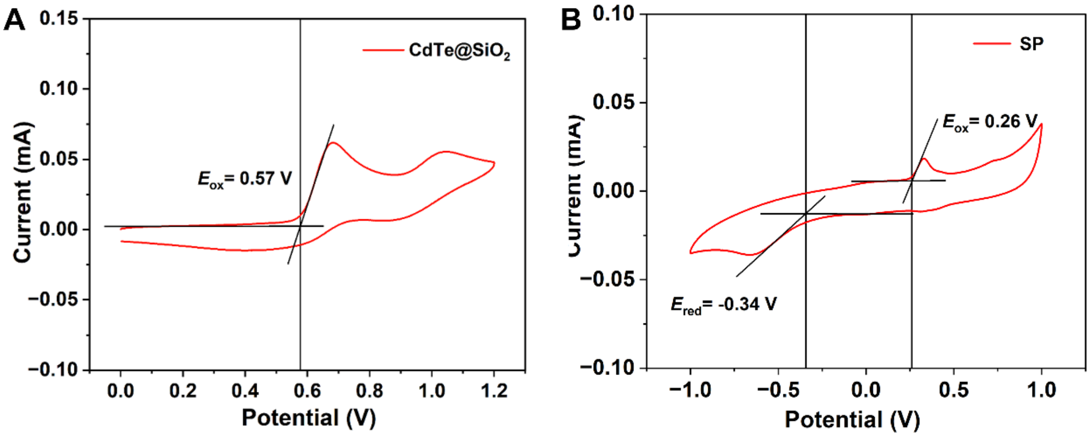

Figure S25. Oxidation-reduction potential detection results. Cyclic voltammetry curves of $CdTe@SiO_2$ A) and SP B).

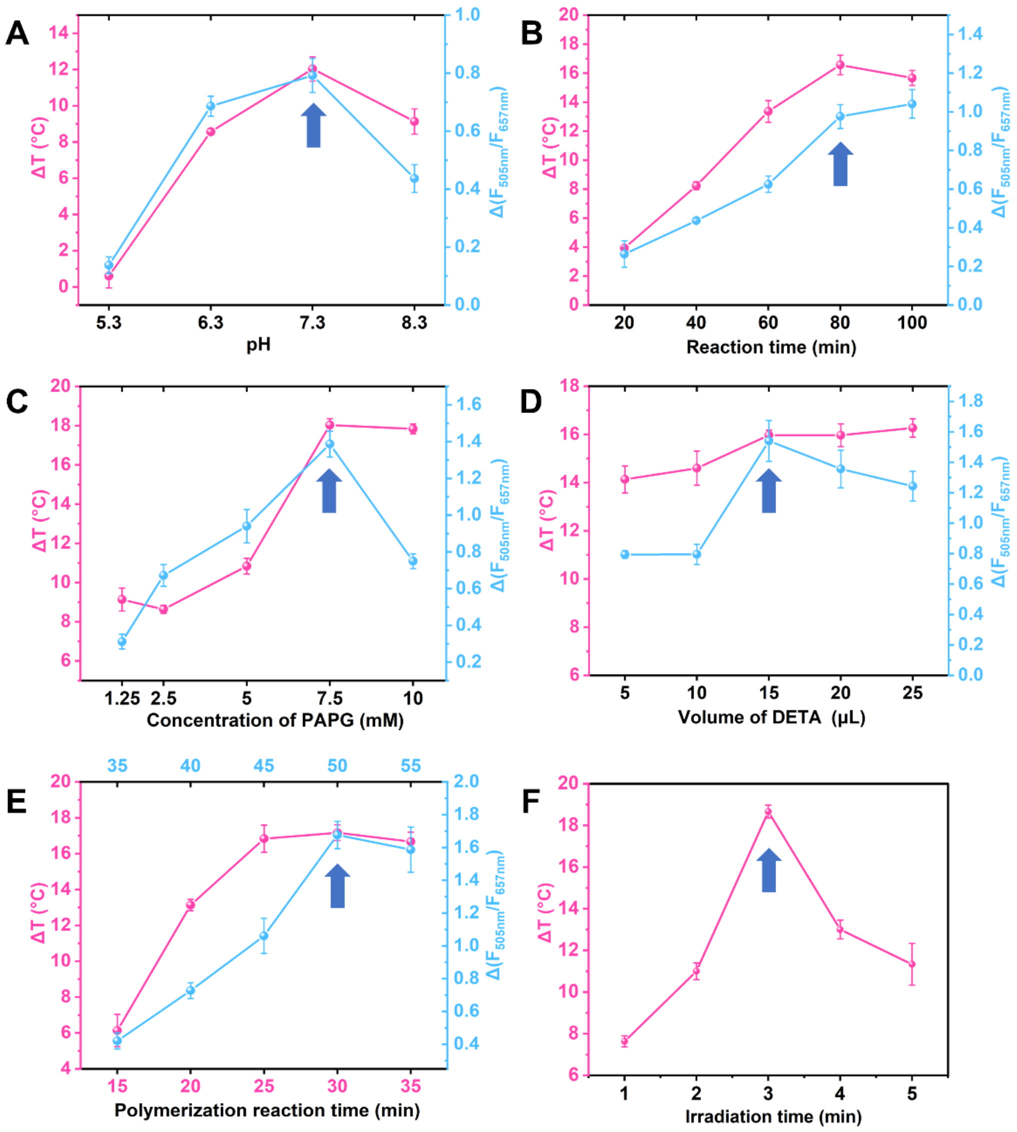

Figure S26. The optimization of 10 mM PIPES buffer pH A), enzymatic reaction time B), PAPG concentration C), volume of DETA (20%, *v*/*v*) ethanol solution D), polymerization reaction time E), and laser irradiation time F). $\Delta(F_{505nm}/F_{657nm})$ and ΔT are the fluorescence ratio variation and the temperature variation between the experimental group (with 80 U L^{-1} β-Gal) and the blank group (without β-Gal). Initial reaction conditions used to optimize the first condition (10 mM PIPES buffer pH) are as follows: the concentration of PAPG is 5 mM, enzymatic reaction time is 60 min, the volume of DETA (20%, *v*/*v*) ethanol solution is 20 μL, polymerization reaction time of photothermal and fluorescent assay are 30 min and 50 min, respectively, the volume of $CdTe@SiO_2$ is 4 μL, and laser irradiation time is 3 min. The parameters are optimized one by one, and each optimal parameter replaces the initial parameter to continue the optimization of the next parameter. Error bars represent the standard deviation of three independent measurements.

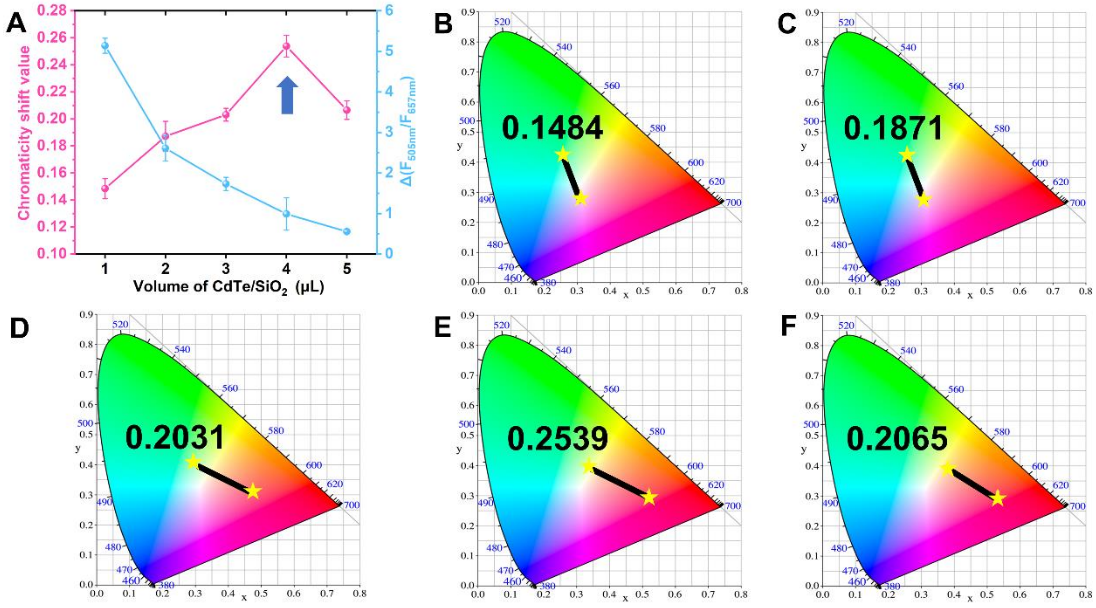

Figure S27. The optimization of CdTe@SiO_2 volume. A) The variation of F_{505nm}/F_{657nm} and the chromaticity shift value versus CdTe@SiO_2 volume. Chromaticity diagram with CdTe@SiO_2 volume of 1 µL B), 2 µL C), 3 µL D), 4 µL E), and 5 µL F), respectively. $\Delta(F_{505nm}/F_{657nm})$ is the fluorescence ratio variation between the experimental group (with 80 U L^{-1} β-Gal) and the blank group (without β-Gal), and the chromaticity shift value is the distance between two coordinates (experimental group and blank group) on the chromaticity diagram. Other conditions: 10 mM PIPES buffer pH is 7.3, the concentration of PAPG is 7.5 mM, enzymatic reaction time is 80 min, the volume of DETA (20%, *v*/*v*) ethanol solution is 15 µL, and polymerization reaction time of fluorescent assay is 50 min. Error bars represent the standard deviation of three independent measurements.

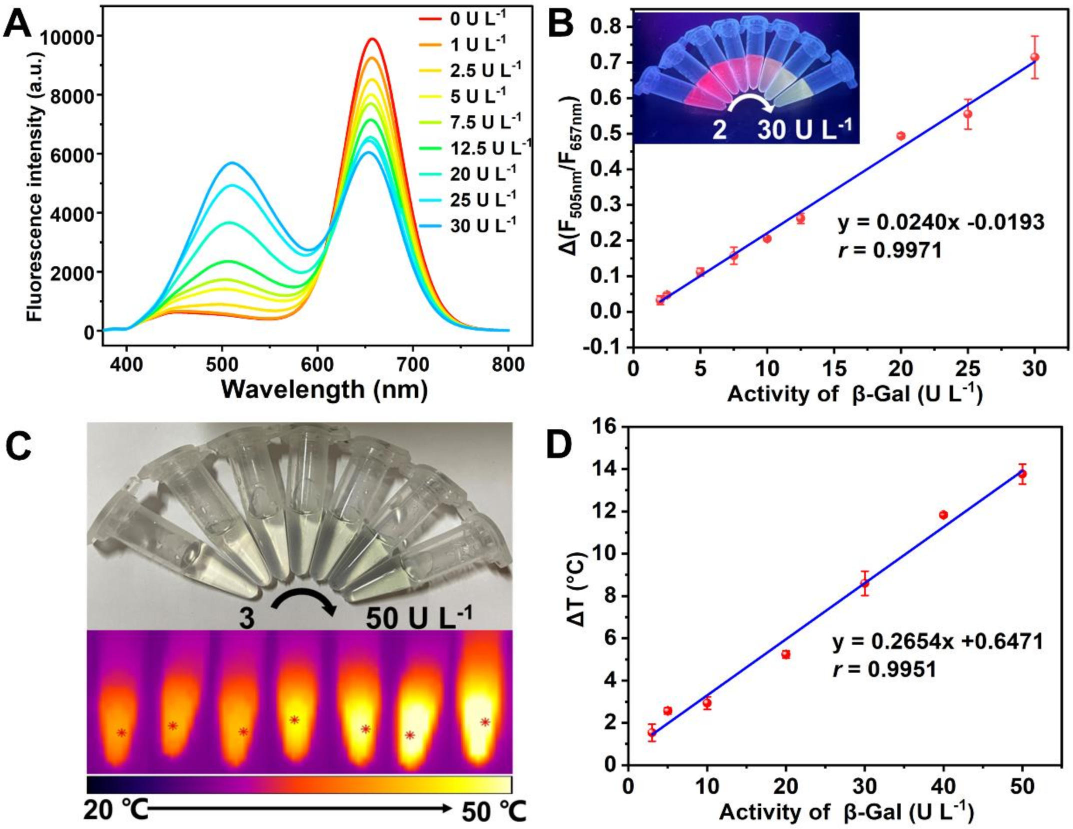

Figure S28. The relationship between β-Gal activity and fluorescence and photothermal. A) The fluorescence spectra of β-Gal activity assay (0–30 U L^{-1}). B) The calibration curve of $\Delta(F_{505nm}/F_{657nm})$ and β-Gal activity (2–30 U L^{-1}). C) The images under daylight and photothermal infrared images at different concentrations of β-Gal (3–50 U L^{-1}). D) The calibration curve of ΔT and β-Gal activity (3–50 U L^{-1}). $\Delta(F_{505nm}/F_{657nm})$ and ΔT are the fluorescence ratio difference and the temperature difference, respectively, between experimental group (with β-Gal) and blank group (without β-Gal). Error bars represent the standard deviations of three repetitive experiments.

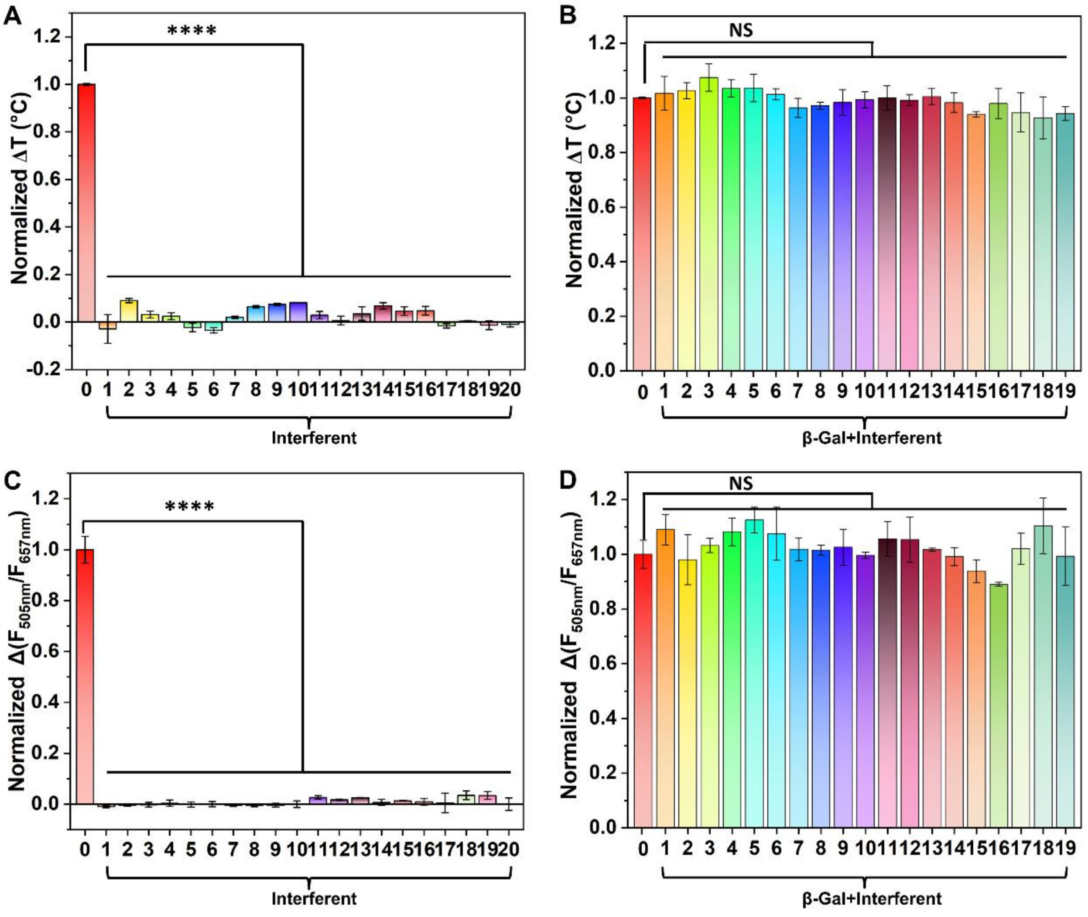

Figure S29. Investigation results of selectivity and anti-interference ability of dual mode method. Selectivity and anti-interference ability of photothermal (A, B) and fluorescence (C, D) sensing for β-Gal detection. 0: 50 U L^{-1} β-Gal; 1–19: Na^{+} (0.1 mM); K^{+} (0.1 mM); CO_3^{2-} (0.1 mM); SO_4^{2-} (0.1 mM); CH_3COO^{-} (0.1 mM); Br^{-} (0.1 mM); glucose (0.5 mM); proline (0.5 mM); glutamic acid (0.5 mM); tyrosine (0.5 mM); chymotrypsin (200 U L^{-1}); trypsin (200 U L^{-1}); lysozyme (200 U L^{-1}); alkaline phosphatase (200 U L^{-1}); ribonuclease (200 U L^{-1}); pepsase (200 U L^{-1}); cysteine (15 μM); glutathione (15 μM); ascorbic acid (15 μM); 20: blank (0 U L^{-1} β-Gal). $\Delta(F_{505nm}/F_{657nm})$ and ΔT are the fluorescence ratio difference and the temperature difference, respectively, between experimental group (with β-Gal) and blank group (without β-Gal). Error bars represent the standard deviations of three repetitive experiments ($^{****}p < 0.0001$; NS: no significant, $p > 0.05$).

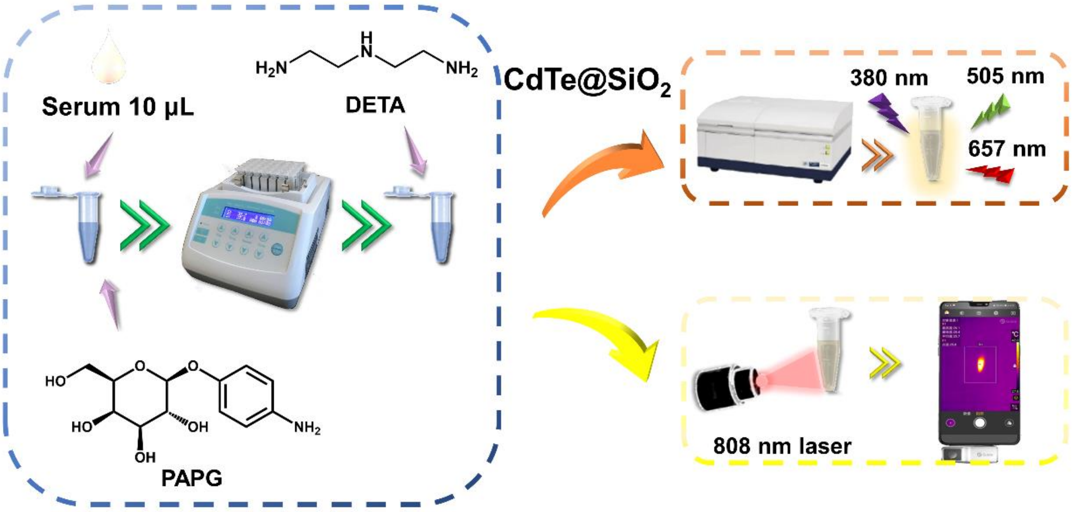

Figure S30. The assay procedure of β-Gal activity of human serum with dual-mode method (photothermal and ratiometric fluorescence assay).

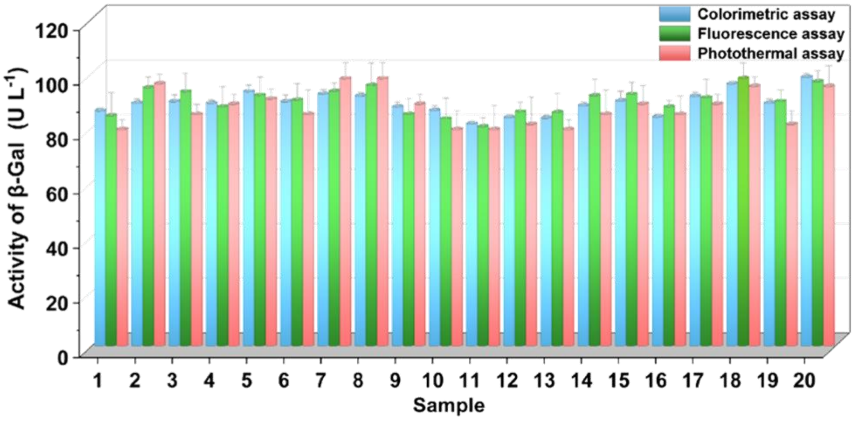

Figure S31. The activity of β-Gal in 20 real serum samples of ovarian cancer patients detected by ratiometric fluorescence assay, photothermal assay, and standard method (colorimetric assay).

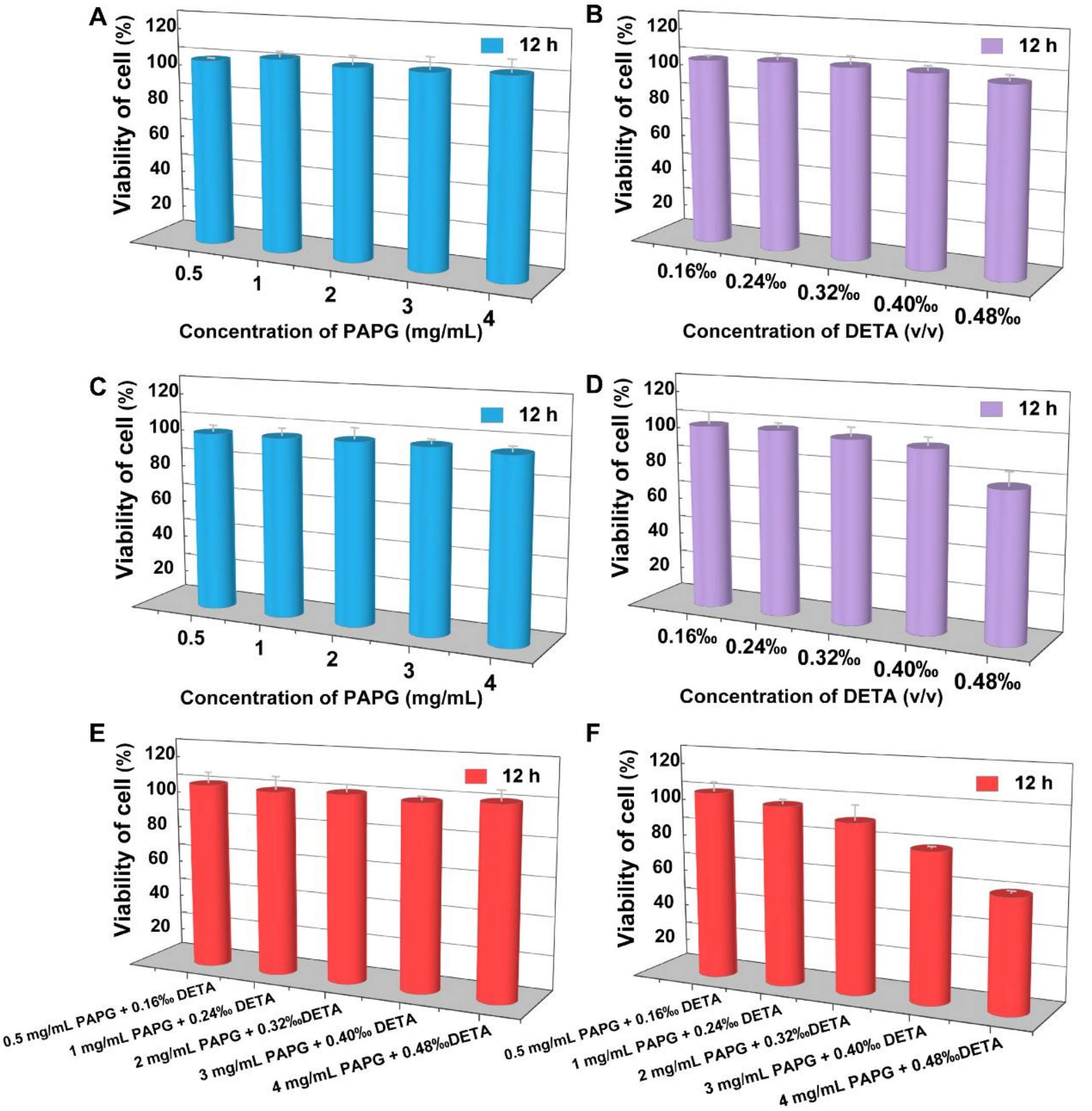

Figure S32. Cell viabilities of Vero after incubation with different concentrations of PAPG A) and DETA B). Cell viabilities of MRC-5 after incubation with different concentrations of PAPG C) and DETA D). Cell viabilities of Vero E) and MRC-5 F) after incubation with mixed solutions of PAPG and DETA in different concentrations. The concentration of PAPG is 0.5, 1, 2, 3, and 4 mg mL^{-1}, respectively. The concentration of DETA is 0.16‰, 0.24‰, 0.32‰, 0.4‰ and 0.48‰ (*v*/*v*), respectively.

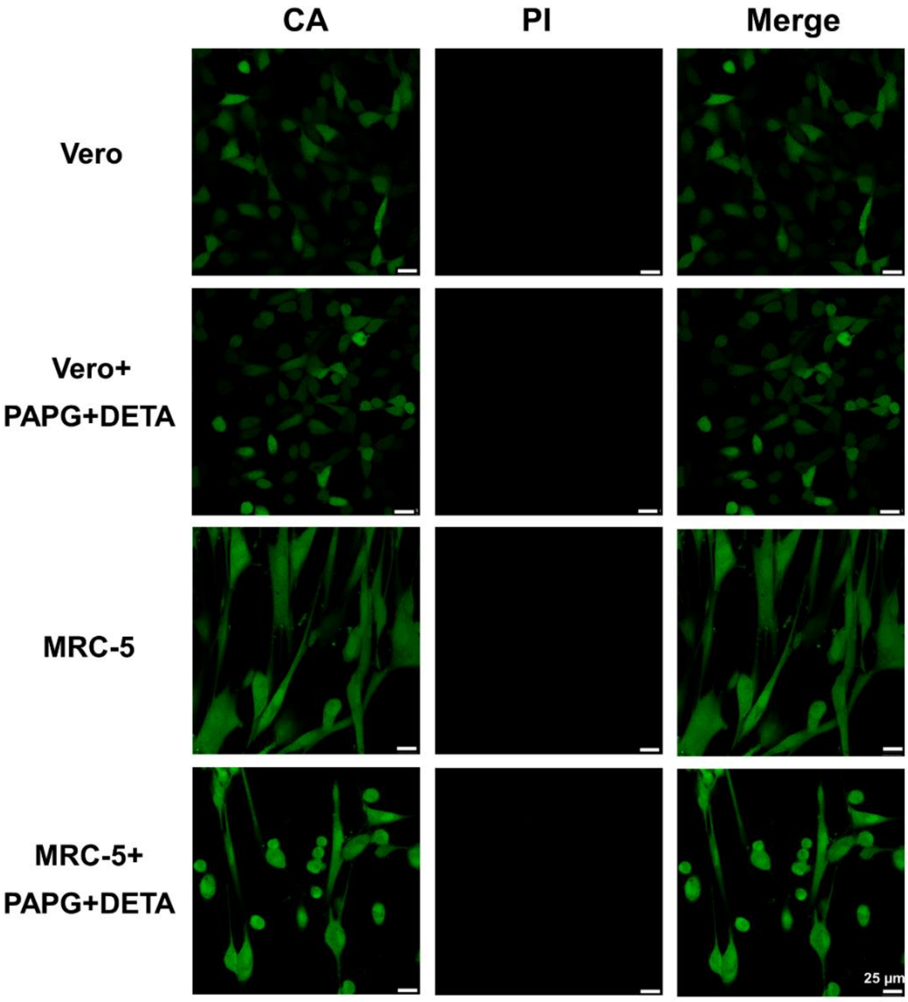

Figure S33. Confocal fluorescence images of Calcein AM and propidium iodide (PI) co-stained Vero and MRC-5 cells after treatments with PAPG and DETA. Green and red colors represent live and dead cells, respectively. The concentration of PAPG is 2 mg mL^{-1}, the concentration of DETA is 0.32‰ (*v*/*v*). The concentrations of Calcein AM and PI are 2 μM and 4.5 μM, respectively. Scale bar: 25 μm.

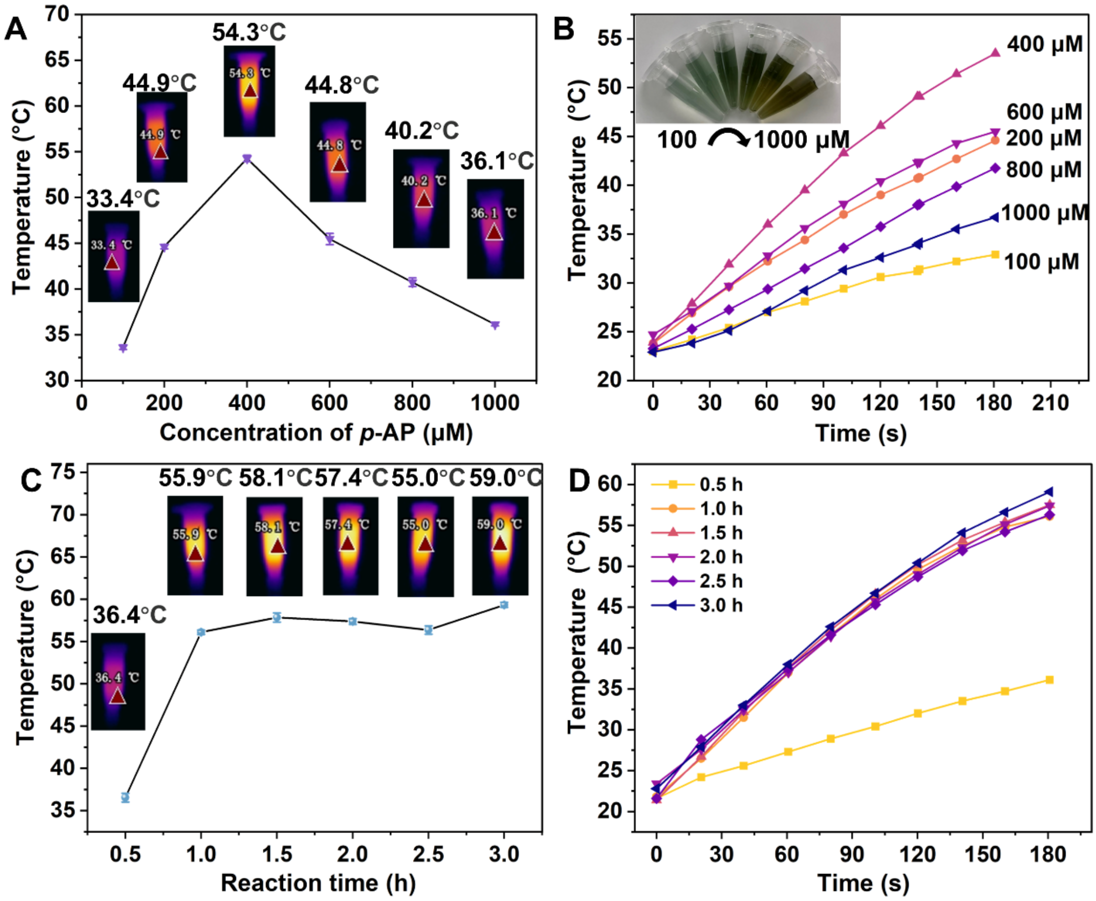

Figure S34. Temperature changes versus different concentrations of *p*-AP (A, B) and polymerization reaction time (C, D). The irradiation time of laser (808 nm, 1.5 W cm^{-2}) is 3 min.

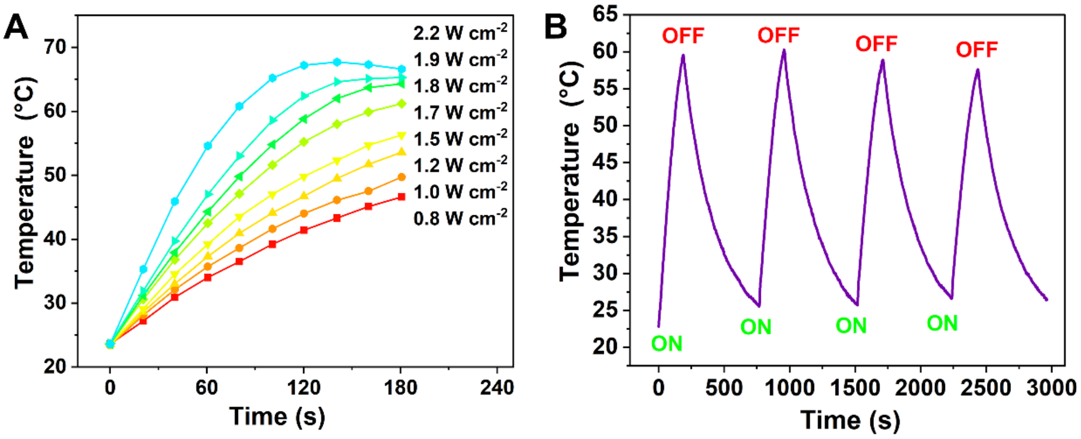

Figure S35. Photothermal property of OM *in vitro*. A) Temperature changes of OM solution under different power laser irradiation. The laser power is 0.8–2.2 W cm^{-2}. B) The temperature profile of OM (synthetic reaction time: 3 h) over four alternative on/off cycles by the 808 nm laser irradiation. The irradiation time of a single laser (808 nm, 1.5 W cm^{-2}) is 3 min.

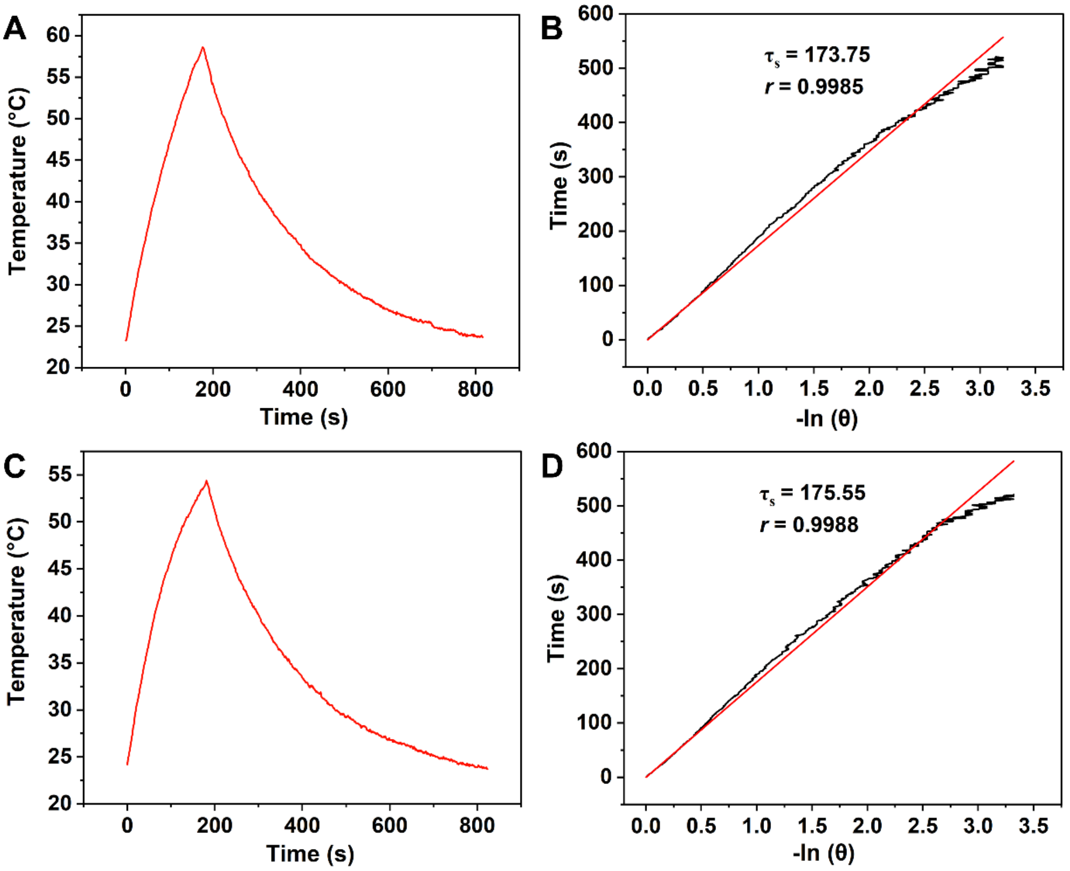

Figure S36. Photothermal conversion ability of ICG and OM *in vitro*. A) Temperature profile of ICG (20 μM) irradiated with 808 nm laser, followed by natural cooling with the turn-off of the laser. B) Determination of ICG (20 μM) time constant (τ_s) through the linear regression of the cooling profile shown in A); according to Eq. 2, the slope of the linear equation is the τ_s value. C) Temperature profile of OM irradiated with 808 nm laser, followed by natural cooling with the turn-off of the laser. D) Determination of OM time constant (τ_s) through the linear regression of the cooling profile shown in C); according to Eq. 2, the slope of the linear equation is the τ_s value.

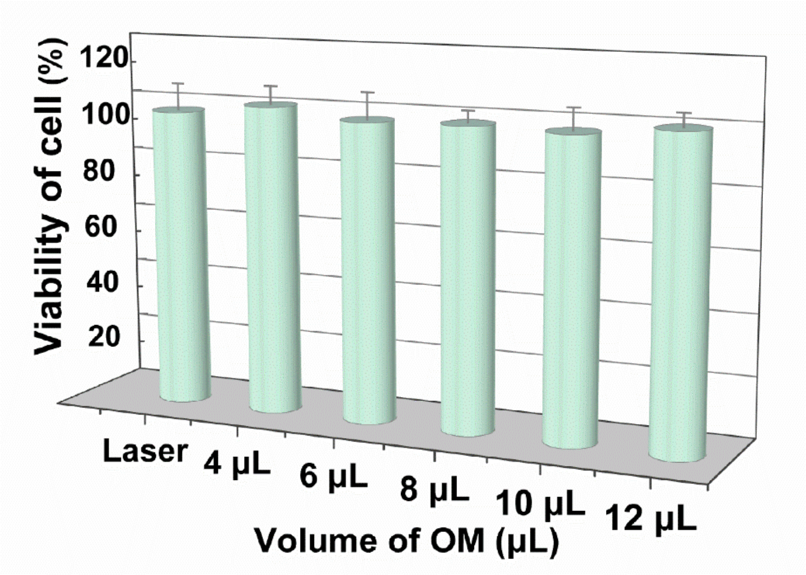

Figure S37. Cell viabilities of Vero cells under laser irradiation and after incubation with different volumes of OM for 12 h. The irradiation time of laser (808 nm, 1.0 W cm^{-2}) is 5 min.

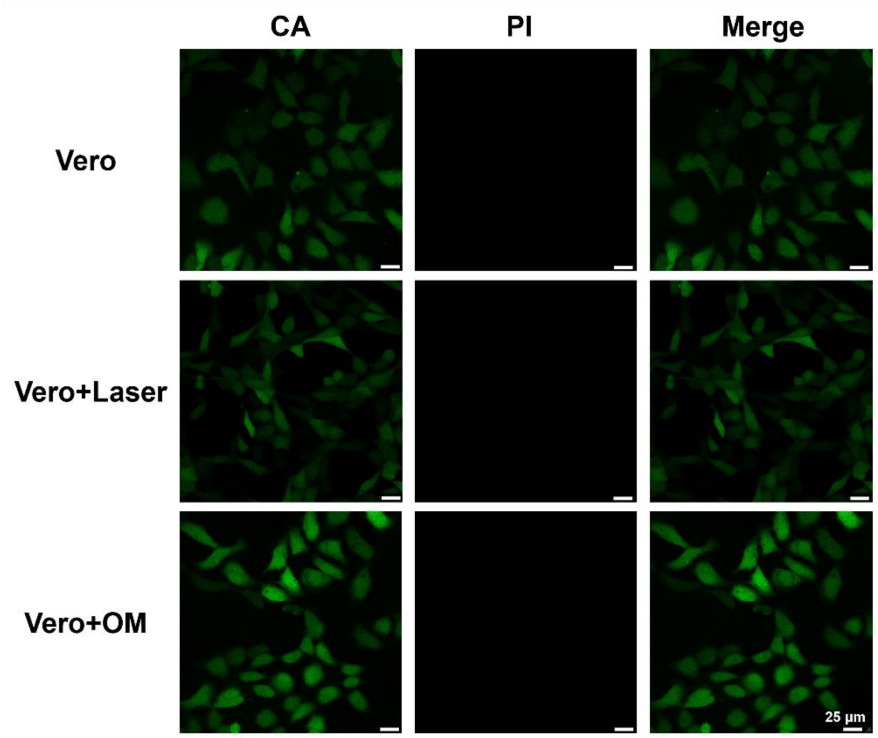

Figure S38. Confocal fluorescence images of Calcein AM and PI co-stained Vero cells after various treatments. Green and red colors represent live and dead cells, respectively. The volume of OM is 25 μL (incubation 12 h) and the laser irradiation time is 5 min (808 nm, 1.0 W cm^{-2}). The concentrations of Calcein AM and PI are 2 μM and 4.5 μM, respectively. Scale bar: 25 μm.

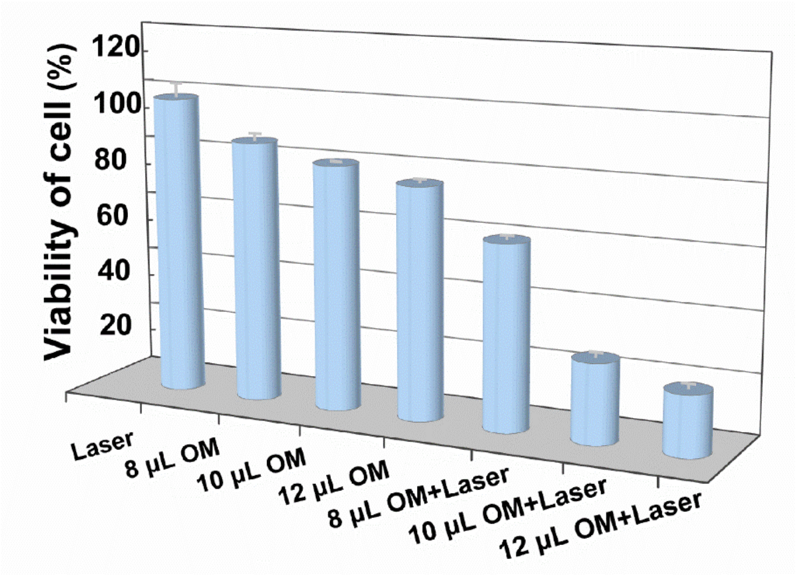

Figure S39. Cell viabilities of SKOV-3 cells after incubation with different volumes of OM for 12 h. The irradiation time of laser (808 nm, 1.0 W cm^{-2}) is 5 min.

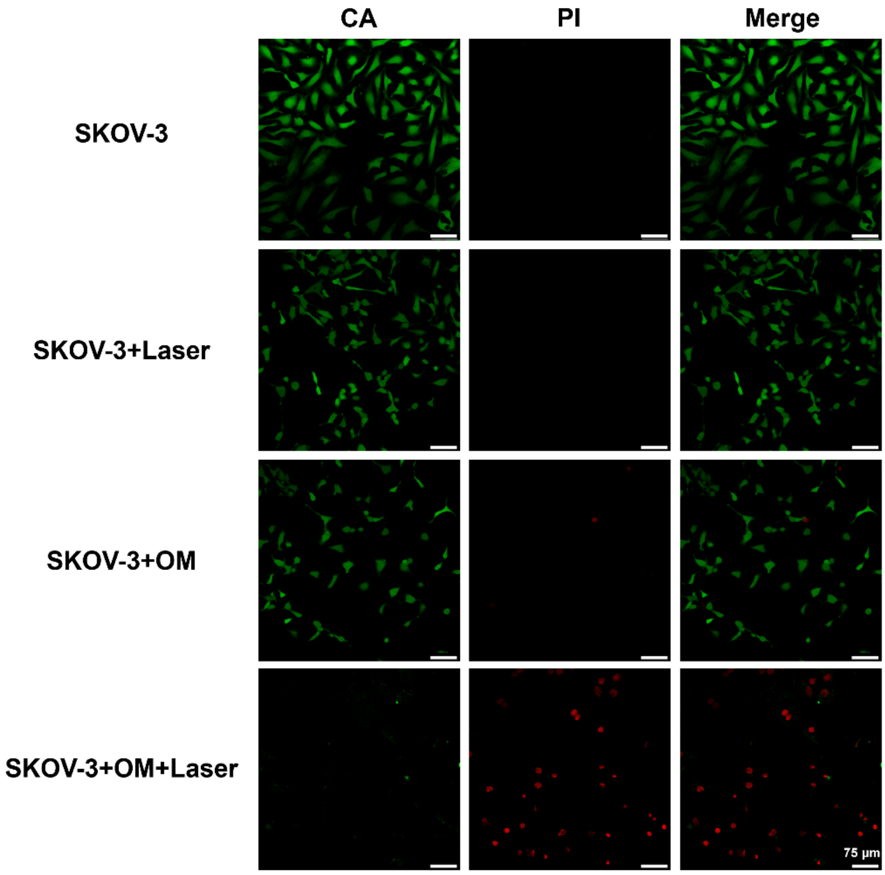

Figure S40. Confocal fluorescence images of Calcein AM and PI co-stained SKOV-3 cells after various treatments. Green and red colors represent live and dead cells, respectively. The volume of OM is 25 μL (incubation 12 h) and the laser irradiation time is 5 min (808 nm, 1.0 W cm^{-2}). The concentrations of Calcein AM and PI are 2 μM and 4.5 μM, respectively. Scale bar: 75 μm.

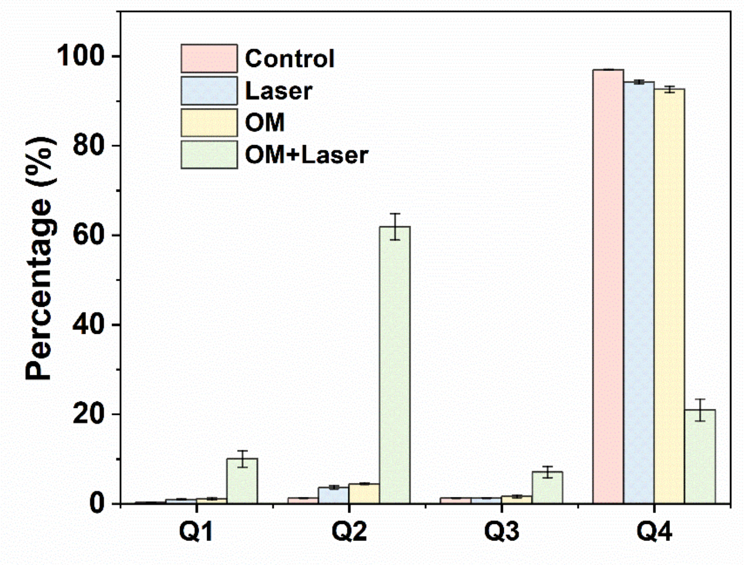

Figure S41. The result of flow cytometry analysis. The columnar statistical analysis plot of SKOV-3 cells treated with control (without any treatment), laser irradiation, OM, OM plus laser irradiation. Q1, Q2, Q3, and Q4 quadrants represent necrotic cells, late apoptotic cells, early apoptotic cells, and live cells, respectively, and the number of cells collected in each group is 10,000. Laser (808 nm, 1.0 W cm^{-2}) irradiation time is 5 min.

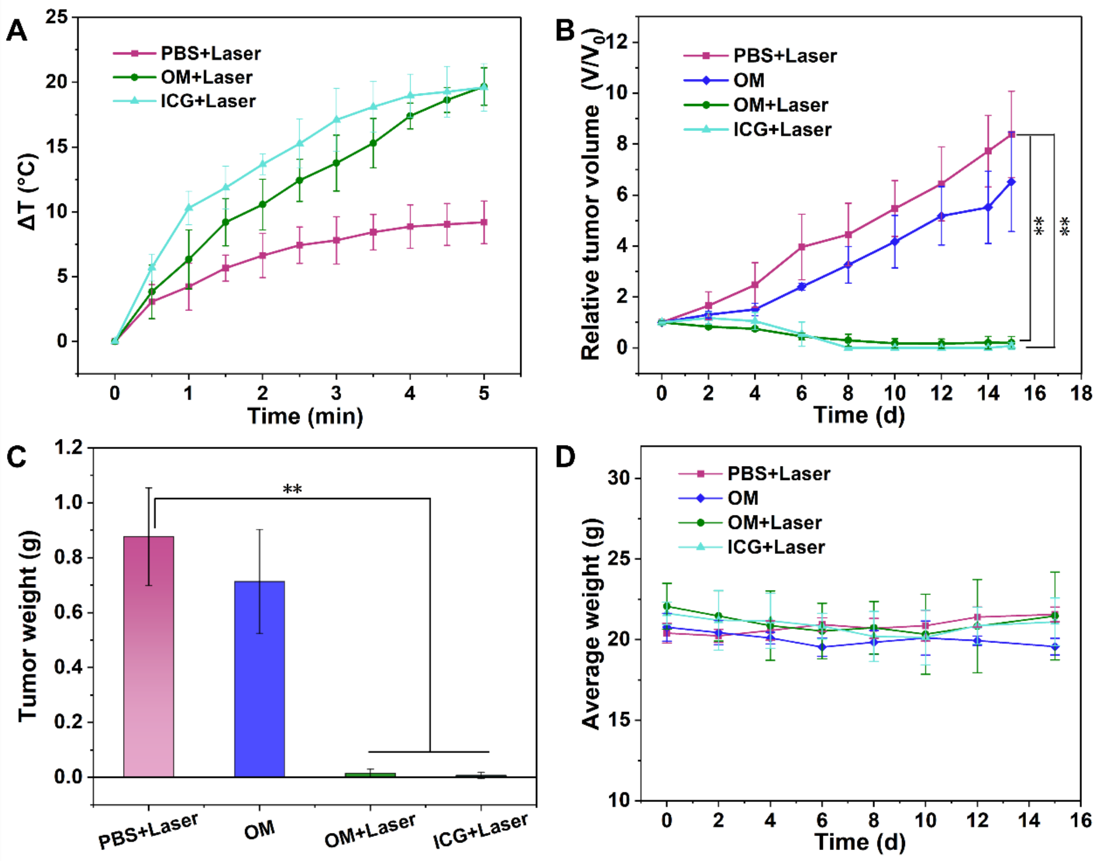

Figure S42. *In vivo* evaluation of the antitumor effect of OM. A) The relationship between temperature change in tumor region of SKOV-3 tumor-bearing mice and laser irradiation time under 808 nm laser irradiation (1.0 W cm^{-2}, 5 min). B) The growth curve of tumor in each group within 15 days. C) The weight of tumor in each group after 15 days of treatment ($^{**}p < 0.01$). D) The weight change curve of mice in each group within 15 days.

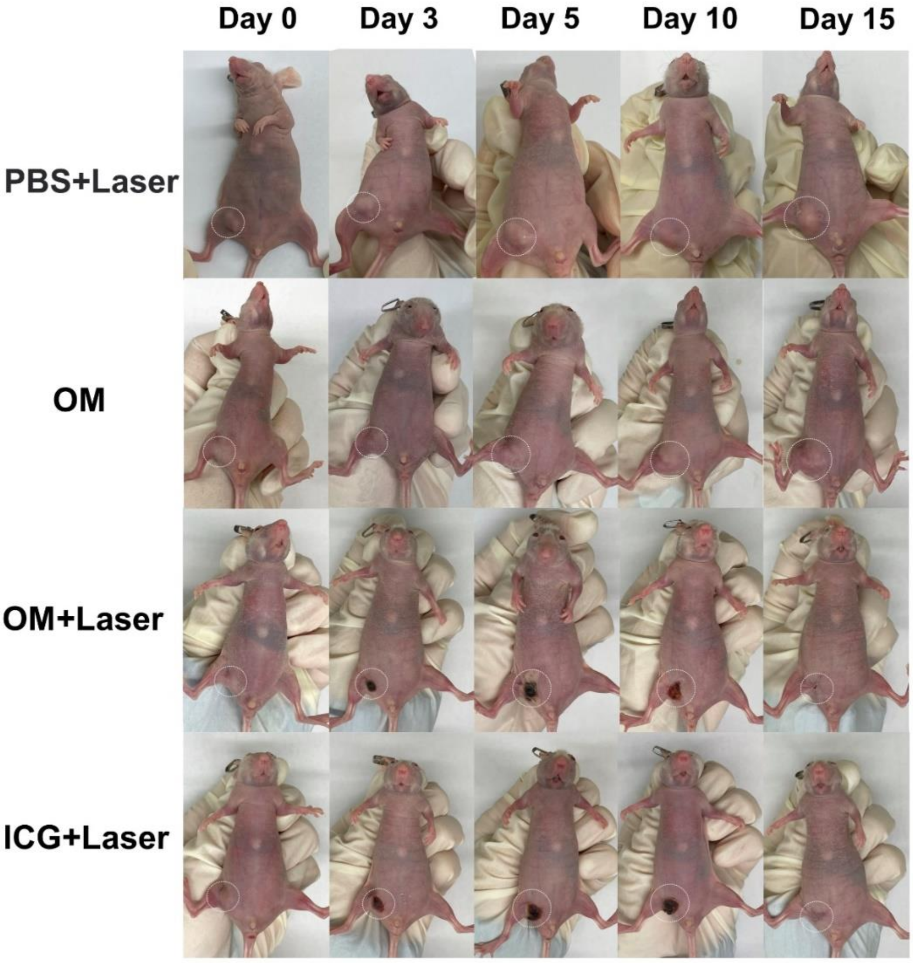

Figure S43. Representative digital photographs of tumor-bearing mice after different treatments.

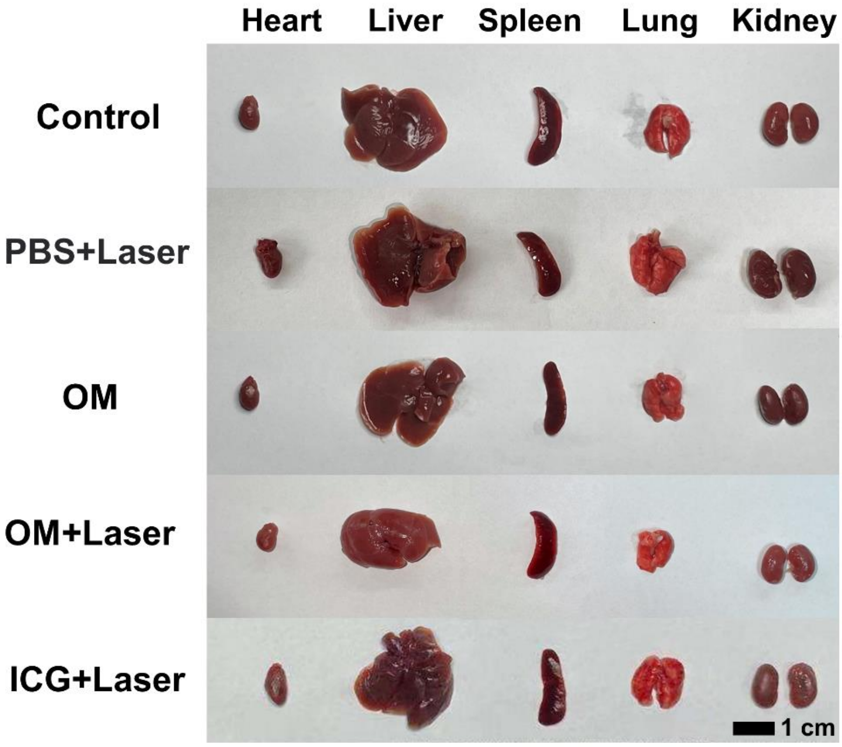

Figure S44. Photographs of the major organs (heart, liver, spleen, lung, and kidney) of normal nude mice (control) and tumor-bearing nude mice represented by each group at the end of the 15-day treatment.

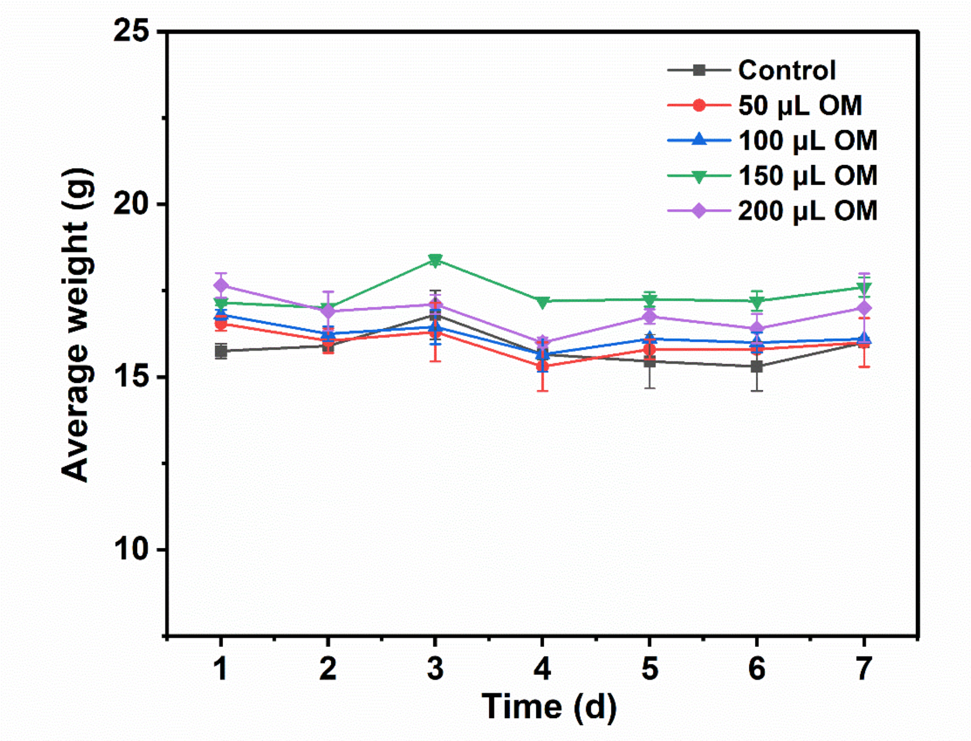

Figure S45. The weight change curve within 7 days of acute toxicity test for each group of nude mice.

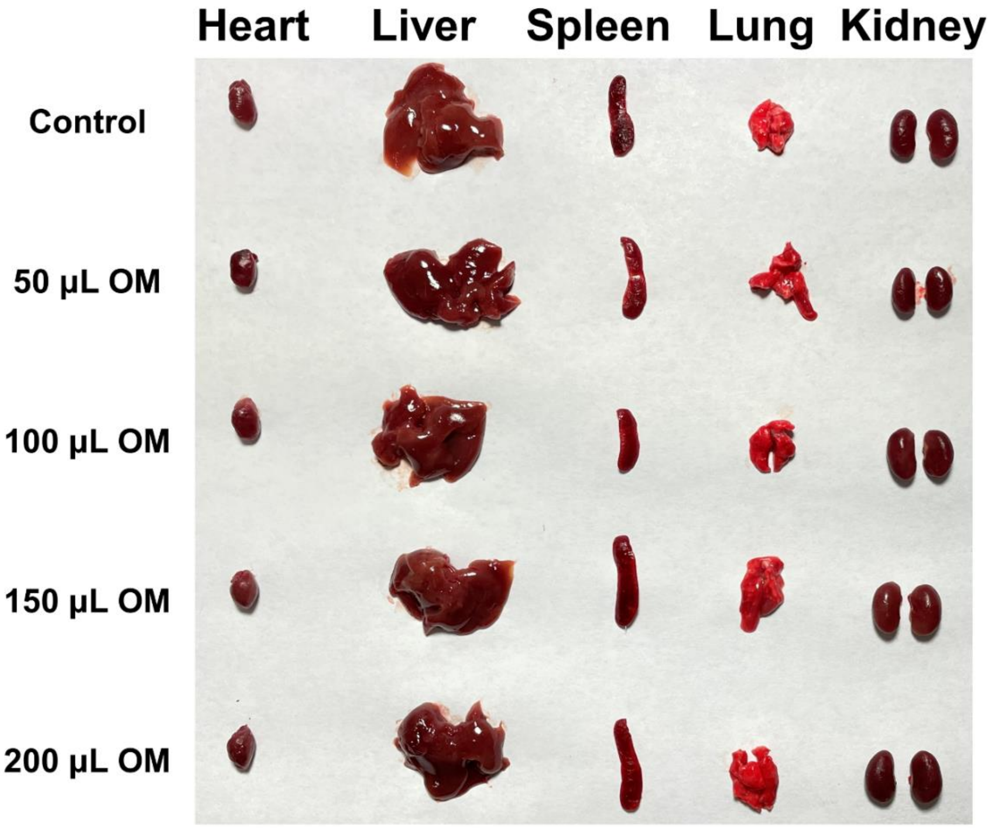

Figure S46. Photographs of the major organs (heart, liver, spleen, lung, and kidney) of nude mice represented by each group at the end of the 7-day acute toxicity experiment.

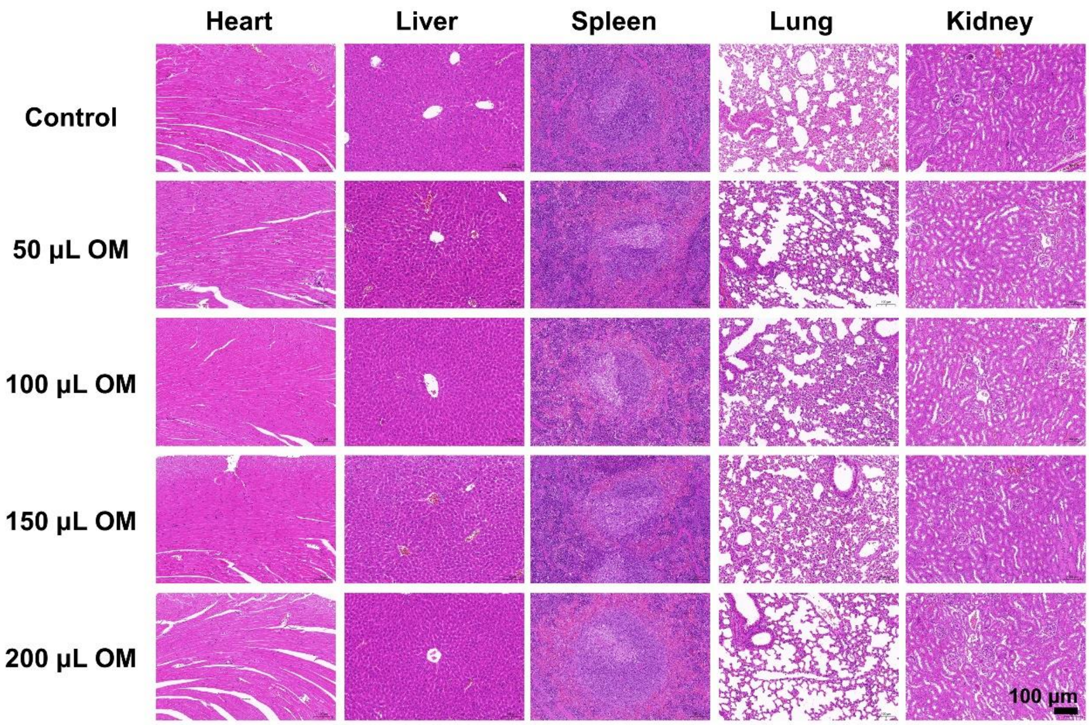

Figure S47. H&E staining of major organs (heart, liver, spleen, lung, and kidney) of nude mice represented by each group at the end of the 7-day acute toxicity experiment.

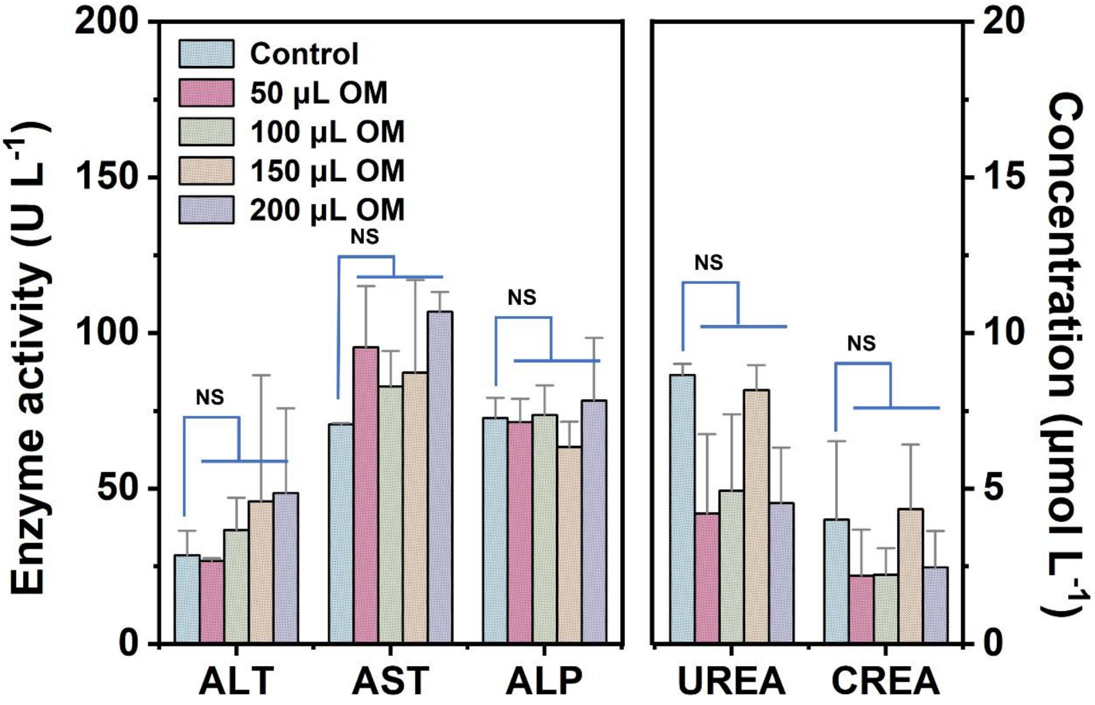

Figure S48. Biochemical analysis results of normal nude mice (control) and treated nude mice in each group, including alanine aminotransferase (ALT), aspartate aminotransferase (AST), alkaline phosphatase (ALP), urea, and creatinine (CREA) (NS: no significant, $p > 0.05$).

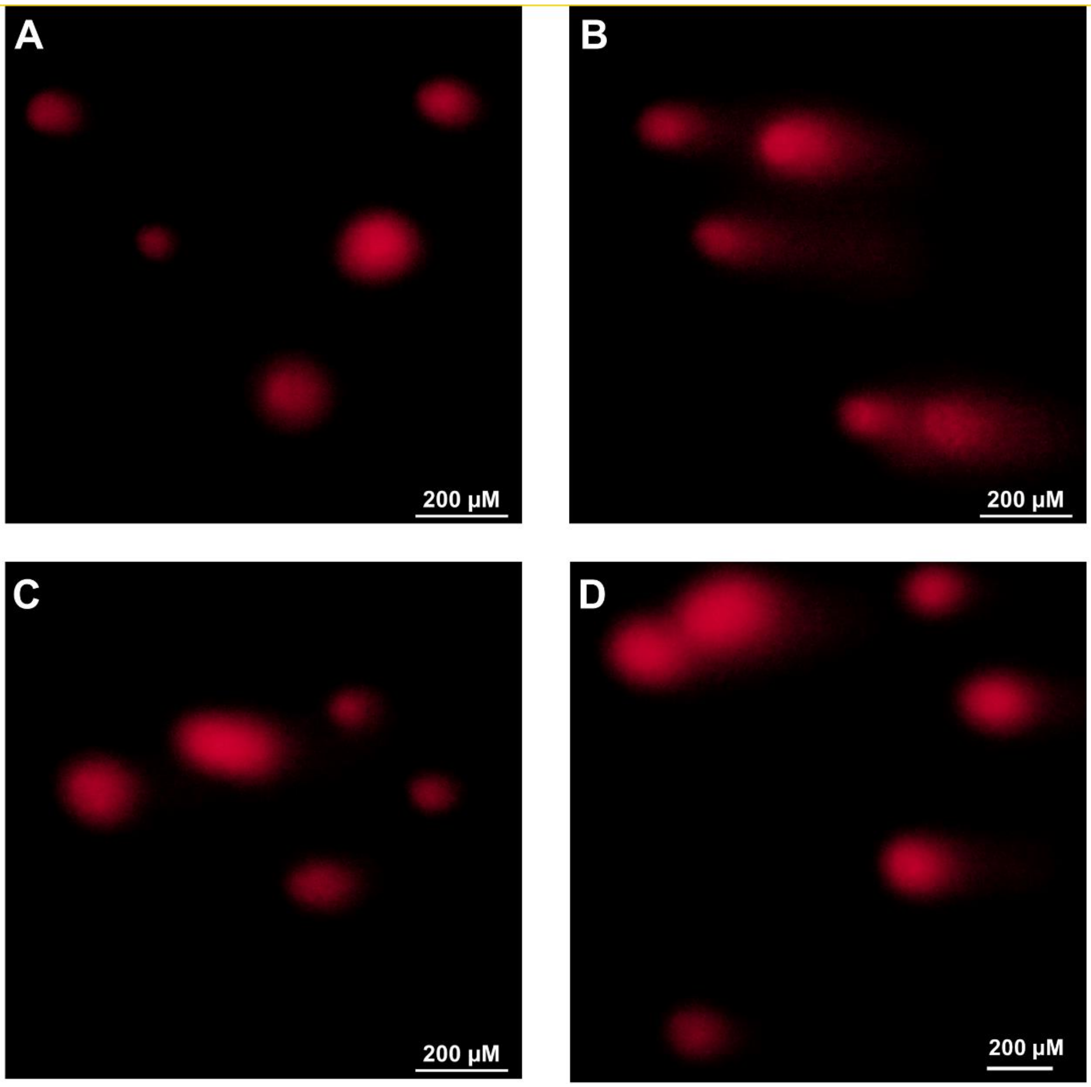

Figure S49. Representative images of comet analysis in MRC-5 cells treated with PBS A), 0.005 μg mL^{-1} MMS B), 40 μL OM C), and 60 μL OM D). (MMS: methyl methanesulfonate).

Table S1. Calculation data of A_3E from S_0 to S_1, S_2 and S_3 state.

		Configurations	λ (nm)	*f*
	S_{01}	D→A (66.5 %), D redistribution (18.8 %), A redistribution (11.4 %).	687.2	0.3937
A_3E	S_{02}	D→A (51.8 %), D redistribution (46.9 %).	570.6	0.0003
	S_{03}	D→A (18.1 %), A→D (24.1 %), D redistribution (48.9 %), A redistribution (8.9 %).	541.0	0.3113

Calculated with time-dependent density functional theory (TD-DFT) at the level of B3LYP/6-31G. S_{01}, S_{02} and S_{03} denoted the first and third vertical transition from S_0 state to S_1, S_2, and S_3 states, respectively. *f* is the oscillator strength between the ground and excited states. D denoted Donor and A denoted Acceptor.

Table S2. Recoveries of β-Gal spiked in real serum samples by fluorescence assay and photothermal assay. (n = 3).

Method	Sample	Added (U L^{-1})	Detected (U L^{-1})	Recovery (%)	RSD (%)
Fluorescence assay	Sample 1	5	4.62	92.4	1.2
		15	16.35	109.0	6.4
		25	21.97	87.9	5.7
	Sample 2	5	5.18	103.6	2.6
		15	13.20	88.0	6.2
		25	23.21	92.8	2.8
Photothermal assay	Sample 1	5	4.98	99.6	5.7
		25	24.87	99.5	2.3
		40	34.34	85.8	3.3
	Sample 2	5	5.07	101.4	8.6
		25	24.02	96.1	3.1
		40	36.90	92.2	2.7
